# Molecular and morphological characterization of hemoprotozoan infections in imported reptiles in Taiwan

**DOI:** 10.1016/j.ijppaw.2025.101164

**Published:** 2025-11-17

**Authors:** Yen-Chi Chang, Tai-Shen Lin, Wei-Wen Huang, Yi-Hsiang Huang, Cheng-Hsin Shih, Ying-Chen Wu, Chiu-Chen Huang, Ter-Hsin Chen

**Affiliations:** a Graduate Institute of Veterinary Pathobiology, National Chung Hsing University, Taichung, 40227, Taiwan; b Research Center for Animal Medicine, National Chung Hsing University, Taichung, Taiwan; c Fiord Animal Hospital, Tainan, 70445, Taiwan; d Graduate Institute of Molecular and Comparative Pathobiology, National Taiwan University, Taipei, 10617, Taiwan; e Department of Post-Baccalaureate Veterinary Medicine, Asia University, Taichung, 41354, Taiwan

**Keywords:** *Hepatozoon*, Histopathology, *Lankesterella*, *Plasmodium*, Phylogenetic analysis

## Abstract

Hemoprotozoa are blood-borne protists with complex life cycles. Despite their high prevalence, diversity of hemoprotozoa in reptiles remains poorly documented. We analyzed blood smears, histopathology, and PCR-amplified 18S rDNA and cytochrome oxidase c subunit I (*COI*) sequences from ten reptiles representing five species imported into Taiwan. In *Varanus macraei,* elongated intraerythrocytic gamonts and hepatic merogonic stages were documented. The near full-length 18S sequence formed a deeply divergent lineage consistent with a novel taxon, for which we propose *Hepatozoon macraei* sp. nov. In *Ctenosaura quinquecarinata* and *Ctenosaura similis*, hemococcidian sporozoites were identified morphologically. Based on 18S rDNA phylogenetic inference, the lineages derived from *C. quinquecarinata* were placed within *Lankesterella*. In addition, all haplotypes from *C. quinquecarinata* clustered with a previously reported *C. similis* lineage based on *COI* phylogenetic inference. In *C. quinquecarinata*, intraerythrocytic *Hepatozoon* were present, and 18S phylogenies formed a well-supported clade closest to *H. ophisauri*. In *Basiliscus plumifrons*, trophozoites and meronts of *Plasmodium* were detected. Analyses of partial 18S rRNA and *COI* sequences each placed the newly generated *Plasmodium* sequence as a separate lineage that did not cluster with available reference sequences. In *Stigmochelys pardalis*, intraerythrocytic gamonts matched *Hepatozoon fitzsimonsi* morphologically and phylogenetically, representing the first record from Taiwan. The current study provides molecular and morphological evidence of multiple hemoprotozoan genera infecting reptilian pets in Taiwan, highlighting the need for further investigation into the diversity, host-pathogen relationships, and potential impacts on native herpetofauna in the global exotic pet trade.

## Highlights

• Three hemoprotozoan genera were identified in imported pet reptiles in Taiwan.
• *Hepatozoon macraei* sp. nov. was proposed from a *Varanus macraei* Böhme and Jacobs, 2001.
• First Taiwan record of *Hepatozoon fitzsimonsi Dias 1953* in a *Stigmochelys pardalis.*
• First *Plasmodium* Marchiafava and Celli 1885 in *Basiliscus plumifrons* Cope 1875 with morphological and molecular description.
• Findings highlight the risk of parasite introduction via the exotic pet trade.

## Graphical abstract

**Figure:**
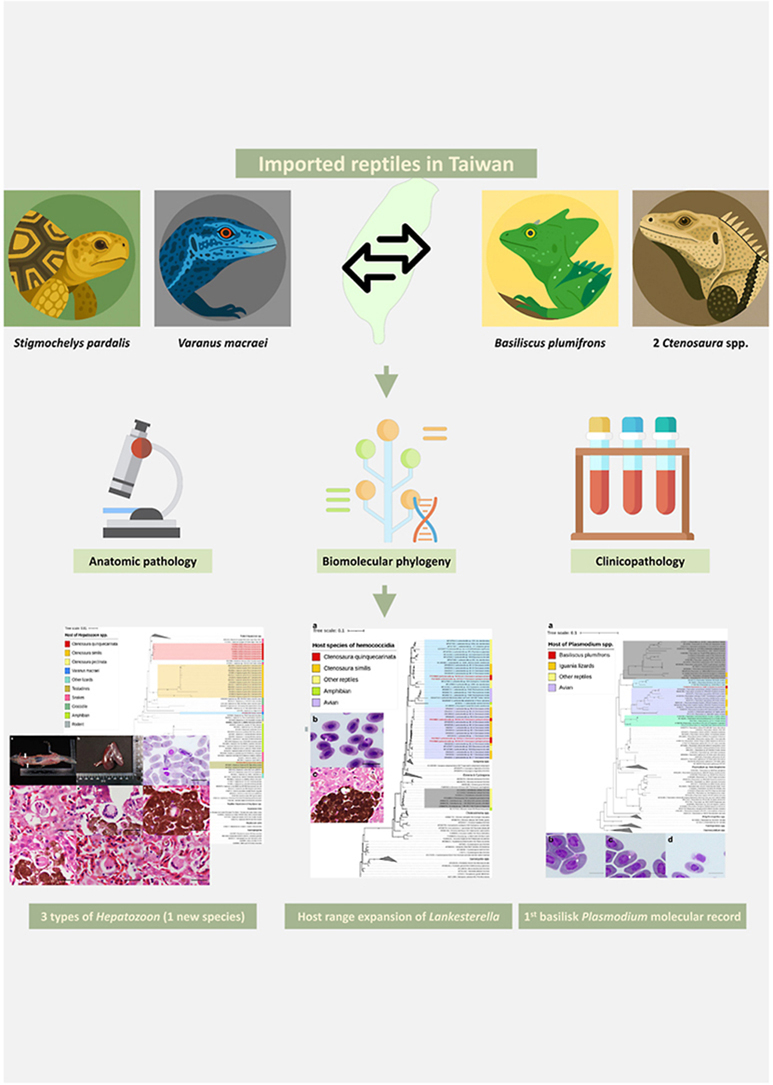

## 1 Introduction

Hemoprotozoa, a diverse group of parasitic protozoa, inhabit the bloodstream of a wide array of vertebrate hosts, including amphibians, reptiles, birds, and mammals (O'Donoghue, 2017; Egan et al., 2021). Despite their prevalence, key aspects of hemoprotozoan diversity, host associations, and ecological roles remain unevenly characterized (Egan et al., 2021; Ndlovu et al., 2024).

*Lankesterella* Labbé,1899 (Apicomplexa: Eucoccidiorida: Lankesterellidae) exhibits a close phylogenetic relationship with the family Eimeriidae (Sailasuta et al., 2011; Megia-Palma et al., 2017; Chagas et al., 2021a). The life cycle typically involves asexual multiplication (merogony) and spore formation (sporogony) within the endothelial cells that line blood vessels and various visceral organs, such as the liver and spleen. The sporozoites are subsequently released into the bloodstream, where they invade erythrocytes. Current evidence indicates that hematophagous invertebrates such as mosquitoes, mites, and leeches act primarily as transport/paratenic hosts, in which sporozoites persist in the invertebrate without further development, and transmission occurs chiefly when vertebrate hosts ingest infected invertebrates (Lainson, 1960; Desser, 1993; Telford, 2016; Chagas et al., 2021b). Bite-mediated transmission has not been demonstrated for *Lankesterella*, although leech involvement has been documented in amphibian and systems (Desser, 1993). Despite increasing research on various lizard species and across different regions, data on host range, pathogenicity, and host-parasite interactions remain limited (Chang et al., 2023; Shabbir et al., 2025). Phylogenetic analyses based on 18S rDNA gene sequences have revealed close relationships among *Lankesterella* parasites infecting amphibians, lizards, and birds, suggesting an ancient origin and diversification of the genus (Megia-Palma et al., 2017; Chagas et al., 2021a; Chang et al., 2023; Saravana Bhavan Venkatachalam et al., 2023; Alves-Coêlho et al., 2025).

The genus *Hepatozoon* Miller 1908 (Apicomplexa: Adeleorina: Hepatozoidae) is a hemogregarine genus that is among the most common, widely distributed, and speciose groups of reptilian hemoprotozoa (O'Donoghue, 2017; Telford, 2016). Species of *Hepatozoon* are obligate heteroxenous organisms, meaning that after fertilization and sporogony in various invertebrate definitive hosts, including ticks, mites, reduviid bugs, sandflies, culicine, anopheline mosquitoes, tsetse flies, lice, fleas, and leeches, which the vertebrate host subsequently ingests, merogony occurs in different tissues, followed by gametogony within the blood cells (Smith, 1996; O'Donoghue, 2017). The pathogenic impact of *Hepatozoon* on the host can be variable at different developmental stages, and the severity of infection in reptilian hosts depends on several factors, such as host swift and parasitic load (Vilcins et al., 2009; Telford, 2016; Bardi et al., 2019). Hematological abnormalities, including anemia, dehemoglobinization, elevated hepatic enzyme concentration, and pathological findings such as multifocal interstitial pneumonia and necrotizing hepatitis have been described (Wozniak et al., 1996; Telford, 2016). While gamont, the life stage that can be easily observed in the blood film of the vertebrate hosts, morphology has historically been used to describe and distinguish different species of *Hepatozoon*, studies in recent years indicate that relying solely on this characteristic is often unreliable and insufficient for accurate species identification due to the variation and the limited morphological features (Smith, 1996; Úngari et al., 2021, 2022). While 18S rDNA remains informative across hemogregarines and related haemococcidians, species delimitation often requires complementary markers chosen for each parasite group and study region (Sloboda et al., 2007; Picelli et al., 2020; Gutierrez-Liberato et al., 2021; Alves-Coêlho et al., 2025).

*Plasmodium* Marchiafava and Celli 1885 (Apicomplexa: Haemospororida: Plasmodiidae) parasites are well-known as the causative agents of malaria in humans (Matta et al., 2018). Human malaria is a major global health concern, causing significant morbidity and mortality, especially in tropical and subtropical regions. Although more than a hundred named reptile *Plasmodium* species have been described, studies on hemosporidians in amphibians and reptiles are still considerably scarcer than those on *Plasmodium* in avian and human, and even less common than research on other reptilian hemoparasites (Matta et al., 2023). For example, by 2019 the MalAvi database had already compiled thousands of lineages of avian *Plasmodium*/*Haemoproteus*, whereas only 12 molecular records of reptilian hemosporidians had been documented, underscoring the disparity in data coverage. Consistent with this gap, new reptile *Plasmodium* lineages continue to be described in recent years, revealing cryptic diversity, and clarifying earlier names (Córdoba et al., 2021; Braga et al., 2025). *Plasmodium* infections in reptiles have been associated with several detrimental effects, including reduced fertility, limited feeding, reduced body mass, and anemia (Ayala, 1978). The high diversity and variability exist among reptile *Plasmodium* species, influenced by factors such as limited host mobility, isolated habitats, and ancient evolutionary history (Braga et al., 2025). Microscopic examination of blood smears has been used to distinguish *Plasmodium* parasites based on the size, shape, and pigment of various life stages (Ayala, 1978; Perkins and Austin, 2009; Matta et al., 2018). However, morphological similarities can obscure distinctions between species, and early descriptions were sometimes insufficient for definitive identification (Braga et al., 2025). Phylogenetic analysis of genetic markers such as the cytochrome *b* (*cytb*) gene, the cytochrome *c* oxidase subunit 1 mitochondrial gene (*COI*)*,* and the 18S rDNA gene offers more precise methods for species identification (Matta et al., 2018; Harris et al., 2019; Córdoba et al., 2021; Braga et al., 2025). However, only a small portion of reptilian *Plasmodium* has genetic data available in public databases (Matta et al., 2018; Braga et al., 2025).

The increasing global trade in animals has led to the widespread movement of species across geographical boundaries, and an increased risk of introducing invasive alien species and novel infectious diseases (Ho et al., 2006; Levings, 2012). In Taiwan, the trade of exotic animals, particularly reptiles, has become increasingly active in recent years (Shiau et al., 2006). The thriving exotic animal market plays a major role in introducing alien species into the native environment. Several amphibians and reptiles have been identified as invasive alien species in Taiwan, with their establishment often linked to the pet trade or intentional releases, including but not limited to *Trachemys scripta elegans* (Wied-Neuwied, 1839), *Kaloula pulchra* Gray 1831, *Anolis sagrei* Duméril and Bibron, 1837, and *Iguana iguana* (Linnaeus 1758) (Lee et al., 2019; Sun et al., 2022). Besides the ecological threats posed by invasive species, imported animals also carry the risk of introducing various infectious diseases, including zoonoses. In a surveillance study targeting pet and rescued smuggled reptiles in Taiwan, *Salmonella* organisms were isolated from 30.9 % of the reptiles investigated. *S*. Typhimurium (Loeffler 1892; Castellani and Chalmers 1919 isolates showed high resistance to multiple antibiotics (Chen et al., 2010). Emerging skin fungal infections caused by *Nannizziopsis guarroi* (J. Cabañes & Abarca) J. Cabañes, Abarca, Guarro, Stchigel & Cano, 2013 have been observed in an *Pogona vitticeps* Ahl, 1927 and an *I*. *iguana* (Linnaeus, 1758), posing a threat to the native reptile population due to the invasive possibility of these reptiles and the lack of predators (Sun et al., 2022). However, despite their highly prevalent nature, parasitic diseases, particularly hemoprotozoan infections, have been overlooked in the surveillance and research of reptile diseases. Despite its conservation status, little is known about the pathogens or infectious diseases affecting this species. This study aims to identify and characterize hemoprotozoan infections in reptiles imported into Taiwan using integrated morphological, histopathological, and molecular methods.

## 2 Materials and methods

### 2.1 Animals and sampling

Between August 2024 to February 2025, 5 individuals of *Ctenosaura quinquecarinata* (Gray, 1842) (CQ1 to CQ5), 2 *Ctenosaura similis* (Gray, 1831) (CS1 and CS2), 1 *Varanus macraei* Böhme and Jacobs, 2001(VP), 1 *Basiliscus plumifrons* Cope 1875 (BP), and 1 *Stigmochelys pardalis* (Bell, 1828) (SP) were referred to Asia University Veterinary Medical Hospital, Taichung City (24.04658° N, 120.68504° E) and Fiord animal hospital Tainan City (23.01590° N, 120.21085° E) with various clinical signs, including depression, anorexia, and dehydration. Blood samples were collected via venipuncture from the ventral coccygeal vein in lizards and the jugular vein in the tortoise for blood film preparation. For body weight <100 g individuals, we used 30-gauge, 0.5 mL insulin syringes; for larger individuals, syringes with 1–3 mL capacity and 25–27-gauge needles were used. For each animal, two to three duplicate thin peripheral blood films were prepared with Superfrost standard microscope slides (Thermo Scientific, PA, USA) immediately after collection from fresh, non-anticoagulated blood and air-dried. For hematology/biochemistry, whole blood was placed in lithium heparin microtubes to minimize EDTA-associated hemolysis reported in reptile (Arikan and Çiçek, 2014). For nucleic-acid workflows, whole blood intended for PCR was collected in EDTA microtubes because EDTA generally preserves nucleotides yield and PCR performance better than heparin, which can inhibit DNA polymerases (Lam et al., 2004; Vaughan-Shaw et al., 2015).

During the follow-up period, all nine lizards died and were discovered either by their owners or by the attending veterinarians. The *V. macraei* (VP) was presented to Fiord Animal Hospital, Tainan with severe emaciation and dehydration; serum biochemistry indicated hyperuricemia. Supportive therapy, including hepatoprotective and gastrointestinal agents, failed to improve the condition, and the lizard was hospitalized for intraosseous fluid administration and assisted feeding. It died after approximately two weeks of hospitalization. The remaining lizards were reported dead by their owners, with nonspecific signs such as anorexia and dehydration. Carcasses of CQ5, CS2, and VP were stored at 4 °C and submitted to the Graduate Institute of Veterinary Pathobiology, National Chung Hsing University for pathological examination. The *S*. *pardalis* case was lost to follow-up after administering the antiprotozoal medication and did not return for further evaluation.

### 2.2 Hemoprotozoan identification

Blood smears were fixed and stained with the Diff-Quik kit (Sysmex, Hyogo, Japan) according to the manufacturer's instructions. For cytologic assessment of visceral stages, tissue wet impression (touch) smears were prepared from freshly sectioned hepatic parenchyma immediately after necropsy of *V. macraei*. The cut surface was gently blotted on absorbent paper, then lightly touched to clean glass slides to avoid cell crushing (Newkirk et al., 2017). Liver smears were air-dried and stained using Diff-Quik and Liu stain (A/B solutions; Baso Diagnostics, Zhuhai, China) following the manufacturer's protocol. Hemoprotozoa were identified by comparing morphological and morphometric data in previous lectures (Telford, 1972, 2016; Cook et al., 2014; Megia-Palma et al., 2017; Walden et al., 2020; Mofokeng et al., 2024). Briefly, hemococcidians were recognized as small fusiform sporozoites within erythrocytes bearing one or more intracytoplasmic refractile bodies, this character is not diagnostic for separating *Lankesterella* and *Schellackia* Reichenow 1919 (Megia-Palma et al., 2017). *Hepatozoon* gamonts were identified as elongate forms with finely granular cytoplasm and a spherical to ovoid nucleus, typically associated with lateral displacement and elongation of the host erythrocyte (Vilcins et al., 2009; Junsiri et al., 2024). Immature *Hepatozoon* gamonts on thin blood films were classified as slender, often arcuate forms with tapered ends, unevenly basophilic, finely granular cytoplasm, and clumped, eccentric nuclei, while mature gamonts were broader with blunt ends and a conspicuous periparasitic space/parasitophorous vacuole, showed evenly light-basophilic cytoplasm and finely stippled, usually central nuclei (Gutierrez-Liberato et al., 2021; Mofokeng et al., 2024). *Plasmodium* intraerythrocytic trophozoites and schizonts were distinguished by triangular to ameboid outlines and the presence of brown-black hemozoin pigment (Telford, 1972). Comparable intracytoplasmic forms lacking hemozoin and occurring in lymphocytes or thrombocytes were recorded as non-erythrocytic *Plasmodium* stages (Córdoba et al., 2021). Slides were examined at 1000 × oil immersion on a BX51 microscope (Olympus, Tokyo, Japan), scanning the monolayer in a zig-zag pattern to avoid field overlap. Infection intensity (parasites per mL) was estimated using a direct counting approach adapted from Carli, in which parasites in 100 non-overlapping fields at 1000 × were enumerated, assuming 100 fields correspond to 0.2 μL of blood; counts were converted to parasites per μL and extrapolated to per mL (De Carli, 2001). Epidemiological descriptors followed Bush et al. (1997). Representative images were captured with a DP21 digital microscopy camera (Olympus, Tokyo, Japan). Morphometrics, including length, width, and area (μm^2^) were measured in Fiji/ImageJ (Schindelin et al., 2012). Measurement was taken along the maximal long and short axes of the parasite or nucleus.

### 2.3 Clinicopathological examination

Heparinized serum samples from the *C. quinquecarinata, C. similis, B. plumifrons, V. macraei,* and *S. pardalis* were analyzed using the VetScan VS2 Analyzer with Avian/Reptilian Profile Plus reagent rotors (Zoetis, Parsippany-Troy Hills, NJ, USA). In the absence of species-specific reference intervals for *C. quinquecarinata, C. similis, and B. plumifrons*, biochemical values were interpreted based on reference data from *I. iguana* as provided in the *Exotic Animal Formulary* 5th edition (Klaphake et al., 2018). For the *V*. *macraei*, reference values from the *Varanus salvator* (Laurenti, 1768) were used.

### 2.4 Pathological examination

Pathological necropsy had only been performed on three specimens: one *C. quinquecarinata* (CQ5), one *C. similis* (CS2), and the *V. macraei* (VP). Major organs—including heart, liver, spleen, lungs, kidneys, skin, brain, gastrointestinal tract, urinary bladder, thigh muscle, gonads, thyroid gland, adrenal glands, and trachea—were examined, photographed, sampled, and fixed in 10 % neutral-buffered formalin for histopathology. Frozen tissue samples were stored at −20 °C until use for subsequent nucleic acid extraction. Fixed tissue samples were trimmed, embedded in paraffin, sectioned at 5 μm, and stained with hematoxylin and eosin (H&E). Slides were evaluated using the same digital imaging system described in the “Clinicopathological examination and hemoprotozoan identification” section.

### 2.5 DNA extraction, amplification, and sequencing

Anticoagulated whole-blood samples were subjected directly to DNA isolation, whereas hepatic tissues were first homogenized with a Biomasher II (Nippi, Tokyo, Japan) before extraction. Genomic DNA was purified with the Tissue/Blood DNA Mini Kit (Geneaid, New Taipei City, Taiwan) following the manufacturer's protocol. *Lankesterella*, *Hepatozoon*, and *Plasmodium* targets were amplified with published primer sets listed in Supplementary Tables S1 and S2 (Keckeisen et al., 2024, Ujvari et al., 2004). To compare host–parasite relationships for the *COI* gene of *Lankesterella*, six archived *C*. *similis* samples from our previous study (Chang et al., 2023) were included in the *COI* PCR. Each 50 μL PCR contained 2 μL of DNA template, Taq DNA Polymerase Master Mix RED (Ampliqon, Odense, Denmark), and 200 nM of each primer, and was run in a MiniAmp Plus thermal cycler (Thermo Fisher Scientific, Waltham, MA, USA). DNA from previously confirmed infections served as positive controls: Lankesterella 18S (GenBank accession number: OR425015), La*nkesterella COI* (**OR427298**; shorter fragment from the same DNA extract), and *Hepatozoon* 18S (**OR425034**). For *Plasmodium*, a positive-control DNA aliquot was provided by another laboratory; it was used **solely to verify primer performance** and **was not included in subsequent analyses.** Nuclease-free water (Qiagen, Hilden, Germany) was used as a negative control in parallel in each PCR run. PCR reaction products were electrophoresed using 1.5 % submarine agarose gels, stained with ethidium bromide, and visualized under ultraviolet trans-illumination. All amplicons were subjected to bidirectional Sanger sequencing (Tri-I Biotech Inc., New Taipei City, Taiwan). Amplicons that produced mixed chromatograms were subsequently cloned with the TOPO® TA Cloning® Kit and One Shot® TOP10 Electrocomp™ *E. coli* (Thermo Fisher Scientific, CA, USA) prior to resequencing. PCR screening was performed under the same PCR conditions. In the positive colonies, the plasmids were purified using the Axygen® AxyPrep™ Plasmid Miniprep Kit (Axygen Biosciences, Union City, CA, USA) according to the manufacturer's instructions and subjected to bilateral Sanger's sequencing with primer set M13F (5′- TGTAAAACGACGGCCAGT-3′) and M13R (5′-ACAGGAAACAGCTATGAC-3′).

### 2.6 Phylogenetic analysis

Following Sanger sequencing, forward and reverse chromatograms were assembled and manually edited into contigs using SeqMan v7.1.0.4 in the Lasergene package (DNASTAR, Madison, WI, USA) (Burland, 1999). Multiple sequence alignments were generated with MAFFT under default settings (Katoh and Standley, 2013). Ambiguously aligned/poorly conserved positions were removed with Gblocks on the NGPhylogeny.fr platform and the curated alignments were used for all downstream analyses (Talavera and Castresana, 2007; Lemoine et al., 2019). Maximum likelihood (ML) phylogenies were constructed in IQ-TREE v3.0.0 (Nguyen et al., 2015). In all datasets, GTR+Γ model was selected as the nucleotide substitution model conducted with the inbuilt ModelFinder (Kalyaanamoorthy et al., 2017). Nodal support was evaluated with 1000 ultrafast bootstrap replicates (Hoang et al., 2018). Bayesian phylogenetic inference was performed in MrBayes via NGPhylogeny.fr with 20,000,000 generations, 2 independent runs, 4 chains per run, sampling every 1000 generations, diagnostics every 5000 generations, and a burn-in fraction of 0.25 before summarizing a majority-rule consensus tree with posterior probabilities (Huelsenbeck and Ronquist, 2001; Lemoine et al., 2019). Resulting trees were visualized and annotated using FigTree v1.4.4 (Rambaut, 2018). Uncorrected pairwise distances were computed as the proportion of nucleotide differences across pairwise-comparable sites with pairwise deletion for gaps and ambiguous bases, and were calculated separately for *Hepatozoon*, *Lankesterella*, and *Plasmodium* under the p-distance model in MEGA X. (Kumar et al., 2018). The alignments for *Hepatozoon* 18S rDNA consist of 54 sequences and 1515 bp, and 82 sequences and 545 bp, respectively. The 18S rDNA sequences from Haemogregarina Danilewsky (1885); Karyolysus Labbé 1894 were used as outgroups in both alignments. The alignment for *Lankesterella* 18S rDNA consists of 41 sequences and 1481 bp. The 18S rDNA sequences from *Isospora* Schneider (1881) and *Eimeria*.

Schneider, 1875 were used as outgroups. The alignment for *Lankesterella COI* consists of 18 sequences and 851 bp. The *COI* sequences from *Isospora* and *Eimeria* were used as outgroups. The alignment for *Plasmodium* 18S rDNA consists of 26 sequences and 560 bp. The 18S rDNA sequences from *Haemoproteus* Kruse (1890) were used as an outgroup. The alignment for *Plasmodium COI* consists of 36 sequences and 1070 bp. The *COI* sequences from *Haemoproteus* were used as an outgroup.

## 3 Results

### 3.1 Clinical examination

All lizards—*C*. *quinquecarinata*, *C. similis*, *V*. *macraei*, and *B*. *plumifrons*—and the tortoise *S*. *pardalis* exhibited stunted growth and signs of dehydration. The findings from physical examinations and serum biochemistry profiles are summarized in Table 1. Due to insufficient blood volume, biochemical analyses could not be conducted for all five *C. quinquecarinata*. In the *S. pardalis*, hematological analysis was not performed despite the high intensity of *Hepatozoon* gamonts observed in blood smears (5 × 10^6^ parasites/mL) owing to financial constraints imposed by the owner. Hemolysis was observed in the blood samples of both *C. similis* individuals likely contributed to spurious hypoglycemia and electrolyte imbalances. *B*. *plumifrons* exhibited marked anemia and hypoalbuminemia. In the *V. macraei* (VP), no apparent hematological or biochemical abnormalities were found, although its albumin concentration was at the lower end of the reference interval.

**Table 1:** Body condition, intensity of hemoparasites, and serum biochemistry of the reptiles in this study.

Items or status	CQ1	CQ2	CQ3	CQ4	CQ5	CS1	CS2	BP	Reference interval^a^	VP	Reference interval^a^
Dehydration	5–10 %	0–5 %	5–10 %	5–10 %	>10 %	3–5 %	3–5 %	5–10 %	–	>10 %	–
Hemococcidia intensity (/mL)	1 × 10^6^	2 × 10^4^	2 × 10^5^	1 × 10^5^	0	0	6 × 10^4^	–	–	–	–
*Hepatozoon* intensity (/mL)	5 × 10^3^	0	1 × 10^5^	0	0	0	0	–	–	2 × 10^6^	–
*Plasmodium* intensity (/mL)	0	0	0	0	0	0	0	5 × 10^5^	–	–	–
Packed cell volume	24	24	25	28	N/A	35	28	13	25–38 %	25	20–47 %
Aspartate aminotransferase	N/A	N/A	N/A	N/A	N/A	45	39	25	0–97 U/L	18	2–58 U/L
Bile acid	N/A	N/A	N/A	N/A	N/A	<35	<35	<35	2.6–30.3 μmol/L	N/A	- umol/L
Creatine kinase	N/A	N/A	N/A	N/A	N/A	2127	3471	851	174–8768 U/L	N/A	176–1818 U/L
Uric acid	N/A	N/A	N/A	N/A	N/A	5.7	5.0	3.9	0–8.2 mg/dL	9.1	1–12.2 mg/dL
Glucose	N/A	N/A	N/A	N/A	N/A	170	**140↓**	120	169–288 mg/dL	N/A	29–170 mg/dL
Calcium	N/A	N/A	N/A	N/A	N/A	9.3	8.5	8.6	6–18 mg/dL	13.7	9.8–18.2 mg/dL
Phosphate	N/A	N/A	N/A	N/A	N/A	6.5	8.5	5.1	2.5–21 mg/dL	6.0	2.9–8.9 mg/dL
Total protein	N/A	N/A	N/A	N/A	N/A	5.2	5.3	2.1	4.1–7.4 g/dL	6	5.1–9.8 g/dL
Albumin	N/A	N/A	N/A	N/A	N/A	2.2	2.3	**<1.0↓**	2.1–2.8 g/dL	1.4	1.4–3.4 g/dL
Globulin	N/A	N/A	N/A	N/A	N/A	3.0	3.0	–	2.5–4.3 g/dL	N/A	2.0–7.3 g/dL
Potassium	N/A	N/A	N/A	N/A	N/A	1.9	**4.8↑**	3.0	1.3–3 mmol/L	N/A	3.5–6.1 mmol/L
Sodium	N/A	N/A	N/A	N/A	N/A	**143↓**	159	147	158–183 mmol/L	N/A	143–170 mmol/L
Hemolysis	N/A	N/A	N/A	N/A	N/A	2+	2+	–	–	–	–

Values outside the reference range were highlighted in bold font, with arrows indicating the direction of deviation.

CS: *Ctenosaura similis*; CQ: *Ctenosaura quinquecarinata*; VP: *Varanus macraei*; SP: *Stigmochelys pardalis*; BP: *Basiliscus plumifrons*. N/A: Not available.

a Retrieved from Exotic Animal Formulary, 5th Edition (Klaphake et al., 2018).

### 3.2 Identification of parasites and pathological findings

The morphometric data of parasites observed in this study are summarized in Table 2.

**Table 2:** Morphometric measurements of hemoprotozoan blood and tissue stages observed in imported reptiles in this study.

Parasites	Host	Development stage	N	C (μm)	PL (μm)	PW (μm)	PA (μm^2^)	NL (μm)	NW (μm)	NA (μm^2^)	RL (μm)	RW (μm)	RA (μm^2^)
*Lankesterella* sp.	CQ	Sporozoites	78	–	10.15 ± 1.53	4.26 ± 0.76	34.06 ± 8.25	2.91 ± 0.37	1.93 ± 0.37	4.43 ± 1.20	2.80 ± 0.69	2.23 ± 0.44	5.10 ± 2.27
	CS	Sporozoites	4	–	14.86 ± 0.58	2.54 ± 0.30	32.80 ± 4.69	2.76 ± 0.22	2.01 ± 0.41	4.38 ± 1.02	3.41 ± 1.25	1.94 ± 0.40	5.52 ± 2.66
*Hepatozoon* sp.	CQ	Mature gamonts	12	–	15.68 ± 2.22	5.15 ± 1.00	64.84 ± 21.00	9.11 ± 1.27	2.98 ± 0.75	21.41 ± 6.33	–	–	–
*H. macraei* sp. nov.	VP	Immature gamonts	29	–	14.53 ± 0.96	4.10 ± 0.47	46.79 ± 6.05	6.98 ± 0.97	2.88 ± 0.44	15.83 ± 3.31	–	–	–
		Mature gamonts	7	–	15.18 ± 1.06	4.62 ± 0.67	55.48 ± 11.53	7.01 ± 0.36	3.14 ± 0.45	17.24 ± 1.91	–	–	–
		Macromeronts	16	3.54 ± 0.70	25.91 ± 3.78	22.36 ± 3.36	463.19 ± 135.33	–	–	–	–	–	–
		Macromerozoites	12	–	15.04 ± 2.63	4.08 ± 0.44	48.35 ± 11.28	3.66 ± 0.78	2.77 ± 0.36	8.04 ± 2.21	–	–	–
		Micromeronts	11	3.44 ± 1.15	23.94 ± 5.86	21.21 ± 3.93	414.62 ± 188.22	–	–	–	–	–	–
		Micromerozoites	15	–	7.97 ± 1.31	2.27 ± 0.39	14.05 ± 2.81	4.31 ± 1.35	1.74 ± 0.31	5.78 ± 1.81	–	–	–
		Monozoic cyst (MC)	2	2.43 ± 0.31	16.41 ± 3.23	15.58 ± 2.92	204.49 ± 77.93	–	–	–	–	–	–
		MC cystozoites	2	–	14.46 ± 2.02	4.45 ± 0.23	50.72 ± 9.66	2.57 ± 0.32	2.33 ± 0.44	4.76 ± 1.48	–	–	–
		Dizoic cyst (DC)	16	2.50 ± 0.42	18.77 ± 1.62	16.36 ± 2.04	243.09 ± 46.42	–	–	–	–	–	–
		DC cystozoites	10	–	15.22 ± 1.26	5.16 ± 0.76	62.10 ± 13.08	2.92 ± 0.38	2.51 ± 0.28	5.82 ± 1.28	–	–	–
		Trizoic cyst (TriC)	8	3.15 ± 1.10	19.04 ± 3.29	17.29 ± 2.77	264.64 ± 94.50	–	–	–	–	–	–
		TriC cystozoite	3	–	14.03 ± 0.72	4.09 ± 0.26	45.17 ± 4.55	2.78 ± 0.30	2.42 ± 0.27	5.32 ± 1.12	–	–	–
		Tetrazoic cyst (TeC)	5	2.79 ± 0.79	22.10 ± 3.20	19.30 ± 2.26	339.41 ± 89.50	–	–	–	–	–	–
		TeC cystozoite	3	–	16.04 ± 0.67	4.50 ± 0.30	56.74 ± 5.78	3.01 ± 0.15	2.57 ± 0.21	6.10 ± 0.69	–	–	–
*H. fitzsimonsi*	SP	Immature gamonts	8	–	14.05 ± 1.22	5.09 ± 0.47	56.20 ± 7.00	4.90 ± 0.76	3.49 ± 0.79	13.62 ± 4.49	–	–	–
		Mature gamonts	43	–	12.45 ± 1.56	4.64 ± 0.47	45.36 ± 7.30	4.12 ± 0.56	2.86 ± 0.43	9.24 ± 1.66	–	–	–
*Plasmodium* sp.	BP	Trophozoites	17	–	3.45 ± 1.25	1.92 ± 0.40	5.38 ± 2.49	–	–	–	–	–	–
		Immature erythrocytic meronts	4	–	4.82 ± 0.69	3.37 ± 0.75	12.69 ± 3.15	–	–	–	–	–	–
		Non-erythrocytic meront	1	–	4.28	2.48	8.35	–	–	–	–	–	–

Measurements are in μm and are presented as mean values ± standard deviations.

N: number of parasites per developmental stage measured; C: capsule thickness; PL: parasite length; PW: parasite width; PA: parasite area; NL: parasite's nuclei length; NW: parasite's nuclei width; NA: parasite's nuclei area; RL: refractile body length; RW: refractile body width; RA: refractile body area; CS: *Ctenosaura similis*; CQ: *Ctenosaura quinquecarinata*; VP: *Varanus macraei*; SP: *Stigmochelys pardalis*; BP: *Basiliscus plumifrons*.

#### *3.2.1* Varanus macraei (VP)

**Fig. 1:**
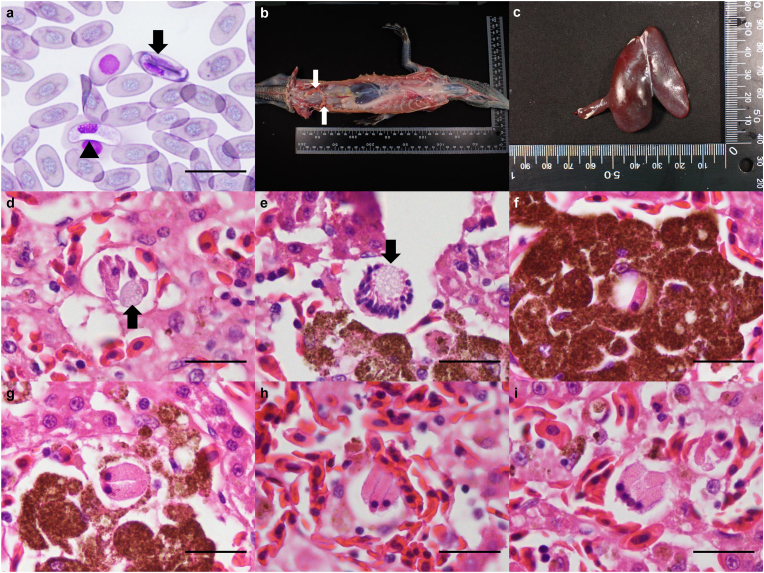
Parasite identification and pathological examination of the *Varanus macraei* Böhme and Jacobs, 2001, specimen VP. a) An immature gamont with basophilic granular cytoplasm (arrow) and a mature gamont with pale cytoplasm and parasitophorous vacuole (arrowhead) of *Hepatozoon macraei* sp. nov. were observed within erythrocytes. The mature gamont was encapsulated by a prominent parasitophorous vacuole. Diff-Quik stain, Bar = 20 μm. b) The lizard was severely emaciated and dehydrated, as indicated by pronounced, diffuse muscle wasting and markedly atrophic fat pads (arrows). c) The liver appeared moderately swollen with a blunt, rounded edge. d) A macromeront containing six spindle-shaped macromerozoites and a centrally located residual body (arrow) was found within the hepatic sinusoidal endothelial cell. H&E, Bar = 20 μm. H&E, Bar = 20 μm. e) A micromeront containing approximately 20 small, palisade-arranged micromerozoites surrounding a foamy residual body (arrow), forming a wheel-spoke pattern. H&E, Bar = 20 μm. f) A monozoic cyst containing a single elongated cystozoite with eosinophilic granular cytoplasm and a round hyperchromatic nucleus was observed within a melanomacrophage. H&E, Bar = 20 μm. g) A dizoic cyst, ovoid in shape with a well-developed capsule, enclosing two banana-shaped cystozoites was observed within the melanomacrophage cytoplasm. h) A trizoic cyst, morphologically similar to the dizoic form, encapsulating three slightly tapered cystozoites was observed within the endothelium. H&E, Bar = 20 μm. i) A tetrazoic cyst encapsulating four slightly tapered cystozoites was observed within the endothelium. H&E, Bar = 20 μm.

Peripheral blood smears contained intraerythrocytic hemogregarine gamonts compatible with *Hepatozoon* (Fig. 1a). Immature gamonts were slender, with curved, tapered ends, unevenly dark-basophilic cytoplasm bearing small eosinophilic granules, and clumped eccentric nuclei (Fig. 1a arrow). Mature gamonts were enclosed by a prominent parasitophorous vacuole, broader with curved but blunt ends, had evenly light-basophilic cytoplasm, finely stippled centrally placed nuclei, and consistently displaced the host-cell nucleus (Fig. 1a arrowhead).

The individual died under veterinary care approximately two weeks after admission. At necropsy, the lizard appeared emaciated and dehydrated, with marked muscle wasting and atrophic fat pads (Fig. 1b). The liver was diffusely dark-red, enlarged, and had a rounded edge (Fig. 1c). The lungs showed multifocal to locally extensive dark-red discoloration, and the airways were partly obstructed by yellow-white caseous material.

Diff-Quik and Liu-stained hepatic impression smears and histological sections revealed multiple adeleorine stages: macromeronts with up to six merozoites (Fig. 1d), micromeronts with up to 24 elongated micromerozoites (Fig. 1e), and monozoic to tetrazoic cysts containing large elongated cystozoites (Fig. 1f–i). These stages occurred within sinusoidal endothelial cells or inside hypertrophic melanomacrophages of markedly hyperplastic melanomacrophage centers. A tetrazoic cyst was also present in splenic endothelium.

Multiple necrotizing granulomatous lesions were present in the lungs, composed of abundant fungal elements, including 3–6 μm, thin-walled oval-to-round yeast (singly or in short pseudohyphal chains) and 3–4 μm-wide, parallel-walled, septate, non-branching hyphae.

#### 3.2.2 Identification of new *Hepatozoon* species in *V*. *macraei*

##### 3.2.2.1 Taxonomic summary

*Hepatozoon macraei* sp. nov. Chang & Chen.

Phylum: Apicomplexa Levine 1970.

Class: Conoidasida Levine 1988.

Subclass: Coccidia Leuckart 1879.

Order: Eucoccidiorida L'eger 1911.

Suborder: Adeleorina L'eger 1911.

Family: Hepatozoidae Wenyon 192.

Genus: *Hepatozoon* Miller 1908.

Type-host: *Varanus macraei* Böhme and Jacobs, 2001 (Squamata: Varanidae).

Type-locality: Fiord animal hospital, Tainan City, Taiwan (23°00′56.7″ N 120°12′38.2″ E).

Site of infection: Peripheral blood erythrocytes, liver, and spleen tissues (merogony).

##### 3.2.2.2 Morphological and morphometric analysis

Etymology: The specific epithet “*macraei*” refers to the name of its host species.

Intensity: 2 × 10^6^ parasites/mL blood (Table 2).

Type specimen: Hapantotypes include a Liu' s-stained thin blood smear containing gamonts and a tissue section of liver with cysts and meronts deposited under collection number ASIZ01000047 in the collection of the Biodiversity Research Museum, Academia Sinica (BRMAS), Taipei, Taiwan.

ZooBank Life Sciences Identifier (LSID): The ZooBank LSID is urn:lsid:zoobank.org:act:EC906C2D-A1B7-44 EF-BB54-2A96A9EA3ED6.

Gene sequence: The 18S rDNA gene sequence (1607 bp) obtained from the blood of V. macraei was deposited in GenBank under accession number PV578808.

**Morphological and morphometric analysis** (Fig. 1; Table 2; Supplementary Fig.1)

Developmental stages observed in blood smears and tissue sections are summarized below.

Immature gamont (Fig. 1a arrow, Supplementary Fig. S1b–m): Immature gamonts measured 14.53 ± 0.96 μm in length, 4.10 ± 0.47 μm in width, and 46.79 ± 6.05 μm^2^ in area (n = 29). They were elongate, with one or both ends curved and tapered, and exhibited unevenly dark basophilic cytoplasm containing small eosinophilic granules. The nuclei were oval to elongate, measuring 6.98 ± 0.97 μm in length, 2.88 ± 0.44 μm in width, and a 15.83 ± 3.31 μm^2^ in area, with a clumped appearance, and located centrally or at approximately one-third of the gamont length. Infected erythrocytes showed partial dehemoglobinization and peripheral displacement of the host cell nucleus.

Mature gamont (Fig. 1a arrowhead, Supplementary Fig. S1n–p): Mature gamonts were slightly larger and broader than immature forms, measuring 15.18 ± 1.06 μm in length and 4.62 ± 0.67 μm in width, and 55.48 ± 11.53 μm^2^ in area (n = 7). Both ends were curved but not tapered. The cytoplasm was evenly light basophilic and lacked granules. The nucleus was ovoid to elongate, measuring 7.01 ± 0.36 μm in length, 3.14 ± 0.45 μm in width, and 17.24 ± 1.91 μm^2^ in area, with a finely stippled appearance. Prominent parasitophorous vacuoles (PV) up to 2.1 μm thick were observed (Supplementary Fig. S1n–p open arrows). Infected erythrocytes exhibited marked nuclear displacement.

Macromeront (Fig. 1d): Macromeronts were observed within the endothelial cells, measuring 25.91 ± 3.78 μm in length and 22.36 ± 3.36 μm in width, with an area of 463.19 ± 135.33 μm^2^, with a pale, 3.54 ± 0.70 μm-thick capsule (n = 16). Immature forms exhibited a wheel-spoke arrangement with a central foamy residual body surrounded by up to 12 macromerozoites aligned peripherally in a palisading pattern. In mature forms, micromerozoites were dispersed without an obvious arrangement pattern. Macromerozoites were spindle-shaped to long-ovoid, measured 15.04 ± 2.63 μm long, 4.08 ± 0.44 μm wide, and 48.35 ± 11.28 μm^2^ in area, with hypereosinophilic, vacuolated cytoplasm (n = 12). Their nuclei were centrally located, circular to ovoid, measured 3.66 ± 0.78 μm long, 2.77 ± 0.36 μm wide, and had an area of 8.04 ± 2.21 μm^2^, with coarsely stippled basophilic chromatin.

Micromeront (Fig. 1e): Micromeronts in hepatic sinusoidal endothelial cells were rounded to oval, measuring 23.94 ± 5.86 μm in length and 21.21 ± 3.93 μm in width, and 414.62 ± 188.22 μm^2^ in area, with a pale, 3.44 ± 1.15 μm-thick capsule (n = 11). Immature forms exhibited a wheel-spoke arrangement with a central foamy residual body surrounded by up to 24 micromerozoites aligned peripherally in a palisading pattern. In mature forms, micromerozoites were dispersed without an obvious arrangement pattern. The size of micromerozoites was 7.97 ± 1.31 μm in length, 2.27 ± 0.39 μm in width, and 14.05 ± 2.81 μm^2^ in area (n = 15). The nucleus of the macromerozoites was dense, basophilic, and ovoid to spindle-shaped, measuring 4.31 ± 1.35 μm in length and 1.74 ± 0.31 μm in width, with an area of 5.78 ± 1.81 μm^2^.

Monozoic cyst (Fig. 1f): Only two monozoic cysts were observed, both within melanomacrophages. The cysts had a 2.43 ± 0.31 μm-thick capsule. Cystozoites were elongate with convex ends, eosinophilic, and finely granular, measuring 14.46 ± 2.02 in length, 4.45 ± 0.23 in width, and 50.72 ± 9.66 in area (n = 2). Nuclei were located near the end adjacent to the residual body, round, and hyperchromatic, measuring 2.57 ± 0.32 in length, 2.33 ± 0.44 in width, and an area of 4.76 ± 1.48 μm^2^.

Dizoic cyst (Fig. 1g): Dizoic cysts were predominantly located within the cytoplasm of melanomacrophage cells. They exhibited an ovoid shape and occasionally contained a centrally located, foamy, light eosinophilic to amphophilic residual body. Each cyst was enclosed by a well-developed capsule measuring 2.50 ± 0.42 μm in thickness. Cysts measured 18.77 ± 1.62 μm in length and 16.36 ± 2.04 μm in width, with an area of 243.09 ± 46.42 μm^2^ (n = 16). Inside each cyst, two banana-shaped cystozoites were observed, each slightly tapered at both ends. The nuclei were small, rounded nucleus with dense, basophilic chromatin. The cystozoites measure 15.22 ± 1.26 μm in length, 5.16 ± 0.76 μm in width, and an area of 62.10 ± 13.08 μm^2^ (n = 10). The nuclei of the cystozoites measure 2.92 ± 0.38 μm in length, 2.51 ± 0.28 μm in width, and an area of 5.82 ± 1.28 μm^2^.

Trizoic cysts (Fig. 1h): Except for the number of cystozoites, the trizoic cysts shared morphological characteristics with dizoic cysts. The cysts measure 19.04 ± 3.29 μm in length, 17.29 ± 2.77 μm in width, and an area of 264.64 ± 94.50 μm^2^ (n = 8). The capsule thickness was 3.15 ± 1.10 μm. Each cyst contained three cystozoites, measured 14.03 ± 0.72 μm in length and 4.09 ± 0.26 μm in width, with an area of 45.17 ± 4.55 μm^2^ (n = 3). Nuclei measured 2.78 ± 0.30 μm in length and 2.42 ± 0.27 μm in width, with an area of 5.32 ± 1.12 μm^2^.

Tetrazoic cyst (Fig. 1i): Tetrazoic cysts measured 22.10 ± 3.20 μm in length and 19.30 ± 2.26 μm in width, with an area of 339.41 ± 89.50 μm^2^ (n = 5). Each cyst enclosed four cystozoites and was surrounded by a capsule with a thickness of 2.79 ± 0.79 μm. The cystozoites measured 16.04 ± 0.67 μm in length and 4.50 ± 0.30 μm in width, with a calculated area of 56.74 ± 5.78 μm^2^ (n = 3). The nuclei of the cystozoites were 3.01 ± 0.15 μm in length and 2.57 ± 0.21 μm in width, with an area of 6.10 ± 0.69 μm^2^.

##### 3.2.2.3 Remarks

Multiple *Hepatozoon* species have been documented in *Varanus* Merrem (1820) across different regions:

*Hepatozoon varani* (Laveran, 1905), described from the *V. niloticus* (Linnaeus,1766), this parasite typically produces elongated gamonts about 11–15 μm in length and 2–4 μm in width. Some reports noted two morphotypes in *H. varani*, including a slender form (12–14 × 2 μm) and a broader form (10–12 × 4–5 μm) (Nicolle and Comte, 1906; Cook et al., 2016). The nucleus of the parasite is often not clearly defined in stained blood smears. Gamonts of *H. macraei* sp. nov. (14.53 ± 0.96 × 4.10 ± 0.47 in immature gamonts and 15.18 ± 1.06 × 4.62 ± 0.67 in mature gamonts) fall at or slightly above the upper size range for *H. varani* and tend to be broader than the slender morphotype; nuclear morphology (finely stippled, clearly delimited) contrasts with the indistinct nucleus reported for *H. varani*.

*Hepatozoon camarai* (Dias, 1954), described from the *V. albigularis*, exhibits two distinct gamont shapes. One is a robust, banana-shaped form (∼11.8 × 5.0 μm), and the other is a longer, curved form (∼14.3–18.3 μm long but only 1.3–4.3 μm wide) (Smith, 1996; Netherlands et al., 2015; Cook et al., 2016). The parasite's nucleus in *H. camarai* is irregular in outline and not confined to a single region of the gamont. The size of *H. macraei* sp. nov. gamonts is longer and narrower than the robust form, and broader than long slender form.

*Hepatozoon borreli* (Nicolle and Comte, 1906) recorded from the *V. griseus* in Tunisia, produces slender gamonts only ∼7–8 μm long and ∼2 μm wide(Nicolle and Comte, 1906; Smith, 1996; Cook et al., 2016). The nucleus (∼1–2 μm) is small and sometimes visible at one end of the parasite. *H. macraei* sp. nov. gamonts are substantially larger and stouter, with a larger, centrally placed nucleus.

*Hepatozoon roshdyi* (Ramadan et al., 1996) described (as *Haemogregarina roshdyi*) from *V. griseus* in Egypt, likely belongs to *Hepatozoon* (Ramadan et al., 1996; Smith, 1996; Cook et al., 2016). Gamonts of *H. roshdyi* are extremely elongate and thread-like, measuring 13–20 μm long but only 1.5–2.5 μm wide. The parasite's nucleus (6.0–8.5 × 1.5–2.5 μm) spans much of the gamont's length. Infected erythrocytes exhibited nuclear displacement, longitudinal stretching, and slight dehemoglobinization, but no karyolysis. Exoerythrocytic development was prominent in the lungs and less frequent in the hepatic tissue. The earliest schizont was round, ∼9 μm in diameter, with an eccentric ∼3.6 μm nucleus. Macromeronts were round (∼25 μm) or oval (20–28.4 × 12–19 μm) and contained up to ∼20 round nuclei (2.4–3.6 μm); derived macromerozoites were elongate (9.4–11.4 × 2–3 μm) with central round nuclei. Micromeroschizonts were round to oval (27–36 × 14.4–15.0 μm), producing up to ∼32 micromerozoites (∼5.4 × 2.4 μm) arranged around a residual body; one end of the micromerozoites was less rounded. Gamonts of *H. macraei* sp. nov. are broader and less filiform. Exoerythrocytic stages of *H. macraei* sp. nov. are typically broader and of greater area, although maximum micromeront length in *H. roshdyi* can be greater than in *H. macraei*. Both macro- and micromeront contained less merozoites than in *H. roshdyi.*

*Hepatozoon toddi* (Wolbach, 1914) was reported in *V. niloticus* (Wolbach, 1914; Cook et al., 2016)*.* Gamonts in peripheral blood are elongate and slender, measuring approximately 10.3 × 2.5 μm; a smaller ovoid form of about 6 × 3 μm has also been noted. Cytoplasm is lightly stained and enclosed by a delicate membrane; the parasite nucleus is typically central, and infected erythrocytes show mild lateral displacement of the host nucleus. Schizogony occurs in endothelial cells, being most abundant in the lungs and liver. Early schizonts are round to ovoid with one or a few nuclei (approximately 1.5 × 5 to 7 × 4 μm), and subsequent nuclear division produces two principal output types: a low-output form yielding about 8 (variants 12 or 24) larger merozoites and a high-output form yielding about 64 (variant 32) smaller merozoites. Correspondingly, merozoites from the low-output form measure roughly 14–16 × 3.5–4 μm, whereas those from the high-output form are ∼2 × 6–8 μm with nuclei ∼2–3.5 μm. Cystic stages reflect these outputs: low-output cysts are about 11 × 19–16 × 22 μm, high-output cysts about 13–15 × 21–23 μm, and 32-type cysts average ∼12 × 18 μm; a residual body is commonly present. Thicker-walled cystic structures containing 4–8 larger individuals have also been described. By comparison, *H. macraei* sp. nov. exhibits larger, stouter intraerythrocytic gamonts. Both macro- and micromeront contained less merozoites than in *H*. *toddi*. The cyst of *H*. *macraei* sp. nov. contained up to 4 individuals, while *H*. *toddi* contained 4 to 8.

*Hepatozoon varanicola* (Mackerras, 1961) has been reported in *V. varius* (Scheelings, 2011; Scheelings and Jessop, 2011; Cook et al., 2016). In one study, hematological examinations were performed on Australian reptiles. 12-μm-long by 4-μm-wide *H. varanicola* gamonts were observed in all 33 *V. varius* specimens, despite the lack of molecular information (Scheelings, 2011). The gamonts have a dark-staining nucleus at one pole and cause a lateral displacement of the nucleus of erythrocytes, though the host cell remains intact. *Gamonts of H. macraei* sp. nov. *are slightly longer and broader and the nuclei are often centrally located rather than distinct polar.*

In addition to the *Hepatozoon* species formally described from *Varanus* spp., several infections in *Varanus* spp. have been reported without formal species designation or accompanying morphological measurements. In **Asia,** several studies in Thailand have detected hemogregarine gamonts in *V. salvator* (Salakij et al., 2014; Junsiri et al., 2024, 2025). Intraerythrocytic gamonts were 11.32 ± 0.886 in length and 4.25 ± 0.621 in width (mean ± standard deviation), which is smaller and narrower than gamonts of *H. macraei* sp. nov. (Junsiri et al., 2024). Another Australian species, *Varanus panoptes* Storr 1980, is also reported to harbor *Hepatozoon* (Vilcins et al., 2009). Intraerythrocytic gamonts in *V. panoptes* were measured only ∼10.0–10.6 μm in length and ∼2.5–3.7 μm in width, making them notably smaller and slimmer than *H. macraei* sp. nov.

Regarding species of *Hepatozoon* described or reported in Indonesia, the origin of *V. macraei*, there are only three records, two of which were identified or described infecting the *Varanus komodoensis* Ouwens, 1912, and *Varanus doreanus* (Meyer, 1874), respectively (Gillespie et al., 2000; Putri et al., 2025; Calil et al., 2017). However, no *Hepatozoon* species from this region combines morphological, morphometric, and molecular data, and no prior record exists for *V. macraei* to our knowledge.

##### 3.2.2.4 Molecular remarks

**Fig. 2:**
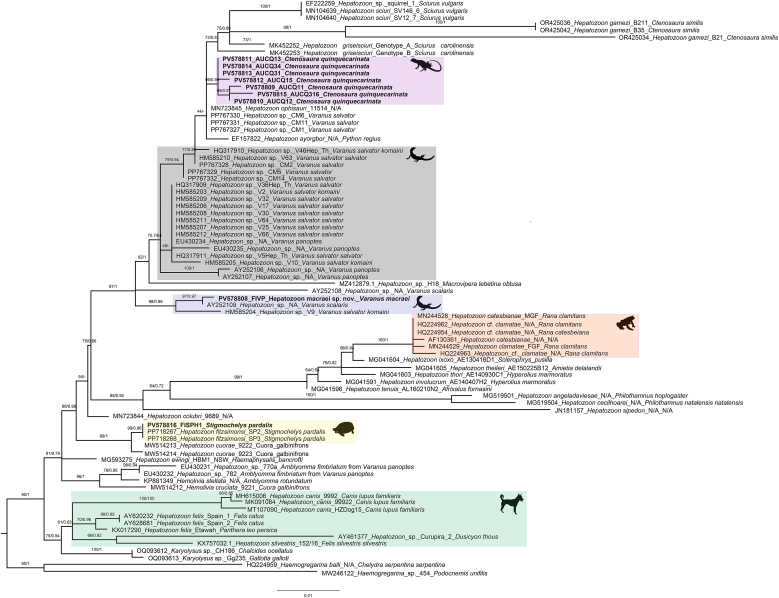
Phylogenetic analysis of *Hepatozoon* Miller (1908) based on 545-bp 18S rDNA sequences. Sequences obtained in this study are highlighted in bold font and are grouped within reptilian and amphibian (orange) *Hepatozoon* and separated from carnivore *Hepatozoon* clade (green shade). Sequences of *Haemogregarina* Danilewsky (1885); *Karyolysus* Labbé 1894 were used as outgroups. Bootstrap values and posterior probabilities are shown close to the nodes. Sequence labels follow th name_haplotype/clone_host species. N/A = Not available in the GenBank source modifier fields. The silhouettes located near the clades indicate the hosts from which the parasites were isolated: a frog for amphibians, an iguana lizard for *Ctenosaura quinquecarinata* (Gray, 1842) (pink shade), a varanid lizard for *Varanus* Ouwens, 1912(gray and blue shades), a tortoise for *Stigmochelys pardalis* (Bell, 1828), and a dog for carnivores. Bayesian inference (BI) and Maximum Likelihood (ML) analysis reveal that sequence derived from V. macraei (GenBank accession number: PV578808) forms a well-supported varanid-associated subclade (blue shade) with two undetermined lineages from varanid hosts (AY252109 from *Varanus scalaris Mertens, 1941* and HM585204 from *V. salvator komaini Nutaphand, 1987*) (ML bootstrap/posterior probability = 98/0.99). All *Hepatozoon* sequences obtained from *C*. *quinquecarinata* (specimens CQ1 and CQ3; PV578809–PV578814) formed a strongly supported monophyletic clade (ML bootstrap/posterior probability = 99/0.96) sister to *H. ophisauri* (Tartakovskii, 1913) from *Pseudopus apodus* (Pallas, 1775) in Iran (MN723845) (pink shade). The sequence from *S*. *pardalis* (PV578816) formed a clade with two *H*. *fitzsimonsi Dias 1953* sequences from the same host species (PP718267 and PP718268) with strong support (ML bootstrap/posterior probability = 99/0.96) (yellow shade).

**Fig. 3:**
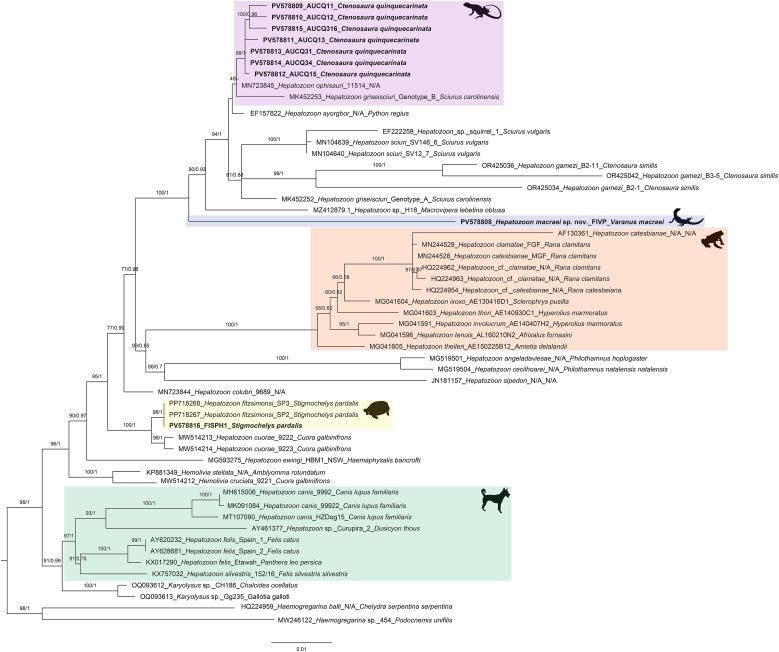
Phylogenetic analysis of *Hepatozoon* Miller (1908) based on 1515-bp 18S rDNA sequences. Sequences obtained in this study are highlighted in bold font and are grouped within reptilian and amphibian (orange) *Hepatozoon* and separated from carnivore *Hepatozoon* clade (green shade). Sequences of *Haemogregarina* Danilewsky (1885); *Karyolysus* Labbé 1894 were used as outgroups. Bootstrap values and posterior probabilities are shown close to the nodes. Sequence labels follow the format: accession number_scientific name_haplotype/clone_host species. N/A = Not available in the GenBank source modifier fields. The silhouettes located near the clades indicate the hosts from which the parasites were isolated: a frog for amphibians, an iguana lizard for *Ctenosaura quinquecarinata* (Gray, 1842) (pink shade), a varanid lizard for *Varanus* Ouwens, 1912(blue shade), a tortoise for *Stigmochelys pardalis* (Bell, 1828), and a dog for carnivores. Bayesian inference (BI) and Maximum Likelihood (ML) analysis reveal that sequence derived from *Varanus macraei* Böhme and Jacobs, 2001 (GenBank accession number: PV578808) within a reptile *Hepatozoon* clade (blue shade) dominated by iguanid and snake hosts, with a comparatively long terminal branch (≈0.044 substitutions per site in the ML hypothesis) (ML bootstrap/posterior probability = 100/1). All *Hepatozoon* sequences obtained from *Ctenosaura quinquecarinata* (Gray, 1842) (specimens CQ1 and CQ3; PV578809–PV578814) formed a strongly supported monophyletic clade (ML bootstrap/posterior probability = 100/0.96) sister to *H. ophisauri* (Tartakovskii, 1913) from *Pseudopus apodus* (Pallas, 1775) in Iran (MN723845) (pink shade). The sequence from *Stigmochelys pardalis* (Bell, 1828) (PV578816) formed a clade with two *H*. *fitzsimonsi* Dias 1953 sequences from the same host species (PP718267 and PP718268) with strong support (ML bootstrap/posterior probability = 98/1) (yellow shade).

Phylogenetic analysis of the 545-bp 18S rDNA fragment placed the *Hepatozoon* sequence from *V. macraei* (PV578808) within the reptilian *Hepatozoon* clade, forming a well-supported varanid-associated subclade with two undetermined lineages from varanid hosts (AY252109 from *Varanus scalaris Mertens, 1941* and HM585204 from *V. salvator komaini Nutaphand, 1987*) (Fig. 2, ML bootstrap/posterior probability = 98/0.99). This varanid cluster was distinct from other varanid-derived *Hepatozoon* sequences represented in the dataset. Consistently, the uncorrected pairwise distance for the 545-bp fragment indicated minimal divergence between PV578808 and AY252109 (0.002) (Supplementary Table S3). In the 1515-bp 18S rDNA tree, PV578808 fell within a reptile *Hepatozoon* clade dominated by iguanid and snake hosts (Fig. 3). The comparatively long terminal branch leading to PV578808 (approximately 0.044 substitutions per site in the ML hypothesis), together with the uncorrected pairwise distance profile for the long-fragment dataset (minimum distance 0.042 to PV578814 from *Ctenosaura quinquecarinata*) (Supplementary Table S4), indicates marked divergence from its nearest available sequence. No additional varanid *Hepatozoon* sequences of sufficient length were available in this analysis, precluding a direct long-fragment comparison with varanid lineages. In conjunction with the detailed morphological evidence presented here, these results support the description of a new *Hepatozoon* species from *V. macraei*.

#### *3.2.3* *Ctenosaura quinquecarinata* (CQ1–CQ5)

**Fig. 4:**
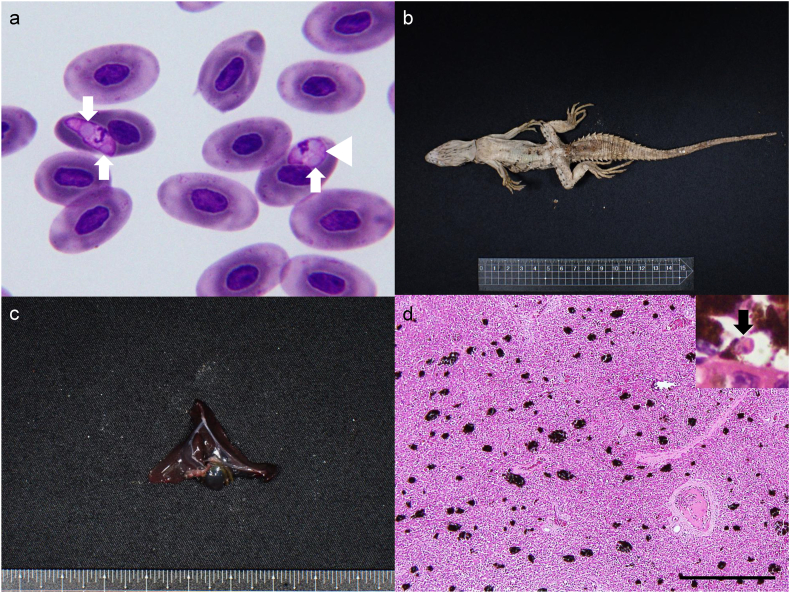
Parasite identification and Pathological examination of the *Ctenosaura quinquecarinata* (Gray, 1842) CQ5. a) Hemococcidian sporozoites were observed within the erythrocytes. The pale-bluish refractile bodies were evident (arrows), and one sporozoite exhibited a curved shape (arrowhead). Diff-Quik, Bar = 20 μm. b) The lizard exhibited severe emaciation and dehydration, as evidenced by marked muscle wasting. c) The liver appeared diffusely dark red. d) Histopathological examination revealed prominent multifocal hyperplasia of melanomacrophage centers, represented by clusters of dark-staining cells distributed among the hepatic parenchyma, accompanied by vascular congestion. H&E, Bar = 500 μm. An ellipsoid, eosinophilic sporozoite with band-shaped nucleus was observed within a melanomacrophage (inset, arrow).

Hemococcidian sporozoites were detected in all *C. quinquecarinata* examined, with intensities ranging from 2 × 10^4^ parasites/mL to 1 × 10^6^ parasites/mL. Sporozoites were erythrocytic and measured 10.15 ± 1.53 μm in length, 4.26 ± 0.76 μm in width, and 34.06 ± 8.25 μm^2^ in area (mean ± standard deviation) (n = 78) They were intracytoplasmic and typically fusiform, with one tapered and one rounded end. Each sporozoite bore one or two pale-blue refractile bodies, possessed amphophilic cytoplasm, and a nucleus positioned toward the tapered end (Fig. 4a). Occasional curved forms with apposed ends (oval to circular outline) were observed (Fig. 4a arrowhead).

At necropsy, CQ5 was severely dehydrated and emaciated, as indicated by pronounced muscle wasting of the extremities and tail (Fig. 4b). No external lesions were observed on the body surface. Necropsy revealed a diffusely dark-brown liver (Fig. 4c). Histopathological examination revealed that melanomacrophage centers were markedly hyperplastic and contained oval-to-fusiform hemococcidian sporozoites (Fig. 4d). Additional lesions included mild, multifocal deposits of amphophilic-to-lightly basophilic, radiating acicular urate tophi within renal tubules and widespread visceral congestion.

**Fig. 5:**
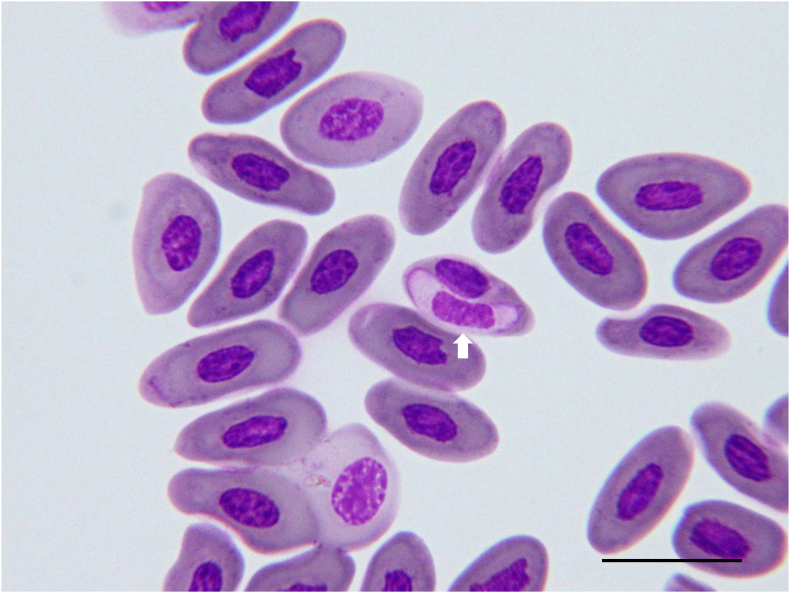
A gamont of *Hepatozoon* Miller (1908) was identified within the erythrocyte of *Ctenosaura quinquecarinata* (Gray, 1842) CQ3 (arrow). Diff-Quik stain, Bar = 20 μm.

Hemogregarine-like gamonts were observed in CQ1 and CQ3 as elongated, intracytoplasmic stages that distorted the host erythrocyte; gamonts measured 15.68 ± 2.22 μm by 5.15 ± 1.00 μm, and area 64.84 ± 21.00 μm^2^) with pale–basophilic cytoplasm and a centrally located, long-elliptical nucleus (9.11 ± 1.27 μm by 2.98 ± 0.75 μm; area 21.41 ± 6.33 μm^2^) (Fig. 5). No hemogregarine-like gamonts were observed in the *C. quinquecarinata* specimens CQ2, CQ4, and CQ5.

#### *3.2.4* *C. similis* (CS1–CS2)

**Fig. 6:**
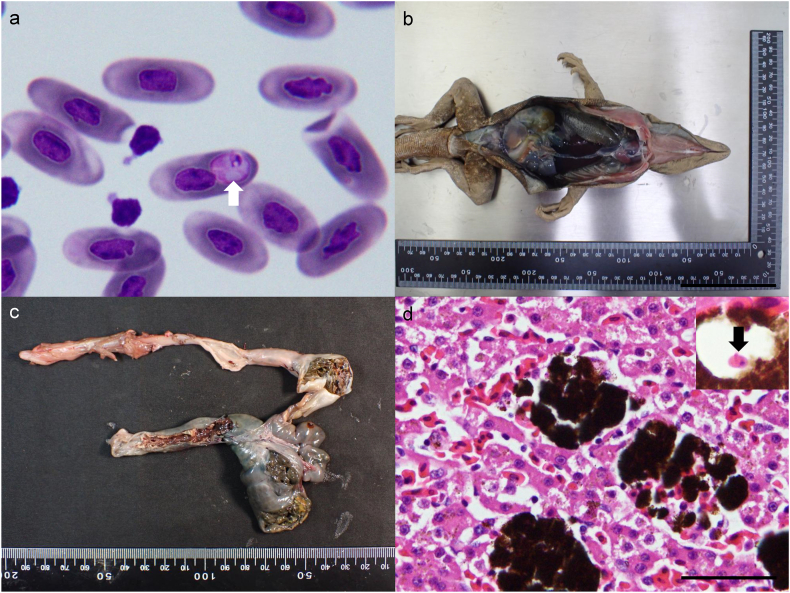
Parasite identification and Pathological examination of the *Ctenosaura similis* (Gray, 1831) CS3. a) Hemococcidian sporozoites were observed within the erythrocytes. The sporozoite exhibited a curved shape, and a pale-bluish refractile bodies were evident (arrow). Diff-Quik, Bar = 20 μm. b) The lizard showed mild dehydration. The lower digestive tract appeared swollen with mottled discoloration, and the liver was dark red. c) The large intestine was markedly distended and filled with turbid, blood-tinged contents. d) Histopathological examination of the liver revealed multifocal, mild to moderate hyperplasia of melanomacrophage centers. H&E, Bar = 50 μm. An ellipsoid, eosinophilic sporozoite with a band-shaped nucleus was observed within a melanomacrophage (inset, arrow).

Hemococcidian sporozoites shared similar morphological features with those observed in *C. quinquecarinata,* with slightly longer and slimmer appearance, measured 14.86 ± 0.58 μm in length, 2.54 ± 0.30 μm in width, and 32.80 ± 4.69 μm^2^ in area (n = 4) (Fig. 6a). The intensity was 6 × 10^4^ parasites/mL.

No hemogregarine-like gamonts were observed in both CS1 and CS2 specimens.

The gross lesions in CS2 specimen revealed emaciation and dark-brown discoloration of liver which were resemble to those observed in CQ5 specimen (Fig. 6b). Additionally, the colonic segment appeared diffusely dark red and contained abundant turbid, blood-tinged to caseous material (Fig. 6c). Histologically, the liver exhibited marked melanomacrophage center hyperplasia with intracellular hemococcidian sporozoites, mirroring the findings in CQ5 (Fig. 6d). Necrotic colitis was characterized by diffuse epithelial ulceration overlain by necrotic debris, fibrin, and rod-shaped bacterial clumps. No protozoan organisms were identified within the necrotic lesions.

#### *3.2.5* *Basiliscus plumifrons* (BP)

**Fig. 7:**
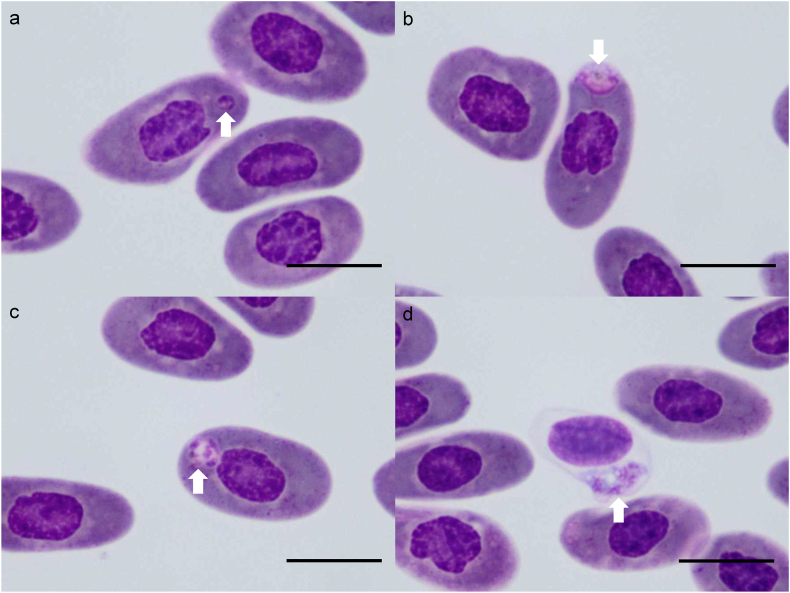
*Plasmodium* Marchiafava and Celli 1885 identification of the *Basiliscus plumifrons* Cope 1875 – BP. a) A tiny intracytoplasmic trophozoite without prominent hemozoin was observed in an erythrocyte (arrow). Diff-Quik, Bar = 10 μm. b) A trophozoite with a hemozoin granule (arrow) was identified within the erythrocyte. Diff-Quik, Bar = 10 μm. c) An intraerythrocytic, multinucleated meront with a hemozoin granule (arrow) was observed in the erythrocytes of BP. Diff-Quik, Bar = 10 μm. d) A multinucleated meront without a hemozoin granule was identified within a thrombocyte (arrow). Diff-Quik, Bar = 10 μm.

Blood films revealed intraerythrocytic stages of *Plasmodium* (Fig. 7a–c). Trophozoites measured 3.45 ± 1.25 μm in length, 1.92 ± 0.40 μm in width, and 5.38 ± 2.49 μm^2^ in area (n = 17), and presented from tiny, without granules (Fig. 7a) to ameboid with measured round, brownish hemozoin granule (Fig. 7b). Immature meronts were polygonal to ovoid, measured 4.82 ± 0.69 μm in length, 3.37 ± 0.75 μm in width, and 12.69 ± 3.15 μm^2^ in area (n = 4) comparable in size to trophozoites, and showed multiple nuclei in lightly basophilic cytoplasm (Fig. 7c). The intensity was 2 × 10^4^ parasites/mL.

By contrast, a larger meront located within a thrombocyte—4.28 μm (length) × 2.48 μm (width), and 8.35 μm^2^ in area—lacked visible hemozoin and was characterized by abundant bluish cytoplasm containing several dark-staining nuclei, which was correlated with non-erythrocytic *Plasmodium* in reptiles in previous study (Córdoba et al., 2021) (Fig. 7d). No additional *Plasmodium* life-cycle stages were observed.

#### *3.2.6* *Stigmochelys pardalis* (SP)

**Fig. 8:**
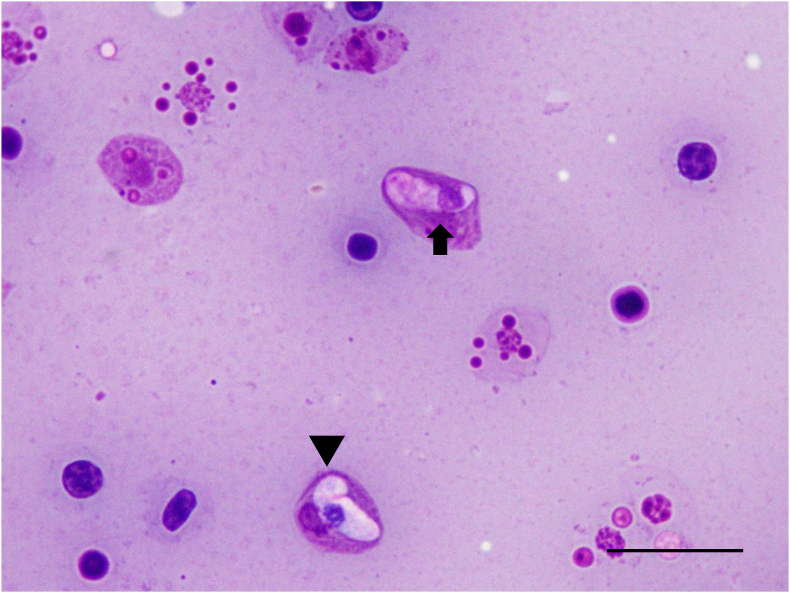
An immature gamont (arrow) and a mature gamont (arrowhead) of *Hepatozoon* Miller (1908) were identified within the erythrocytes of *Stigmochelys pardalis* (Bell, 1828) – SP. Diff-Quik, Bar = 20 μm.

Most erythrocytes were degenerated, characterized by swelling and pale staining, probably due to improper shipping conditions. Intraerythrocytic, elongated, broad with occasional recurved ends hemogregarine gamonts compatible with *Hepatozoon* were present (Fig. 8). Mature gamonts measured 12.45 ± 1.56 μm in length, 4.64 ± 0.47 μm in width, and 45.36 ± 7.30 μm^2^ in area, displayed basophilic cytoplasm and round, finely stippled nuclei (4.12 ± 0.56 μm in length, 2.86 ± 0.43 μm in width, and 9.24 ± 1.66 μm^2^ in area) with parasitophorous vacuole (Fig. 8 arrowhead) (n = 17), while immature forms measured 14.05 ± 1.22 μm in length, 5.09 ± 0.47 μm in width, and 56.20 ± 7.00 μm^2^ in area (n = 8), and were amphophilic to light-eosinophilic with band-shaped, coarsely stippled nuclei (4.90 ± 0.76 μm in length, 3.49 ± 0.79 μm in width, and 13.62 ± 4.49 μm^2^ in area) (Fig. 8 arrow). Both stages frequently displaced the host erythrocyte nucleus peripherally. The intensity was 2 × 10^4^ parasites/mL.

### 3.3 DNA sequencing and genetic phylogenetic analysis

#### *3.3.1* *Ctenosaura quinquecarinata* (CQ1–CQ5)

In phylogenetic trees inferred from both the 545-bp and 1515-bp 18S rDNA datasets, all *Hepatozoon* sequences obtained from *Ctenosaura quinquecarinata* (specimens CQ1 and CQ3; PV578809–PV578814) formed a strongly supported monophyletic clade (ML bootstrap/posterior probability = 99/0.96 for the 545-bp tree and 100/0.96 for the 1515-bp tree) (Fig. 2, Fig. 3). The topology shows a polytomy indicating an unresolved relationship among our lineages, *Hepatozoon ophisauri* (Tartakovskii, 1913) from *Pseudopus apodus* (Pallas, 1775) in Iran (MN723845), and *Hepatozoon griseisciuri* Clark, 1958 from *Sciurus carolinensis* Gmelin, 1788. Despite both hosts being congeners within *Ctenosaura*, the *C. quinquecarinata Hepatozoon* clade is clearly distinct from *Hepatozzon gamezi* Desser (1997) detected in *C. similis* (GenBank: OR425034, OR425036 and OR425042). The uncorrected pairwise distances between sequences obtained in *C. quinquecarinata* ranged from 0.000 to 0.005, while ranged from 0.033 to 0.055 comparing with *H. gamezi* sequences (Supplementary Table S4).

**Fig. 9:**
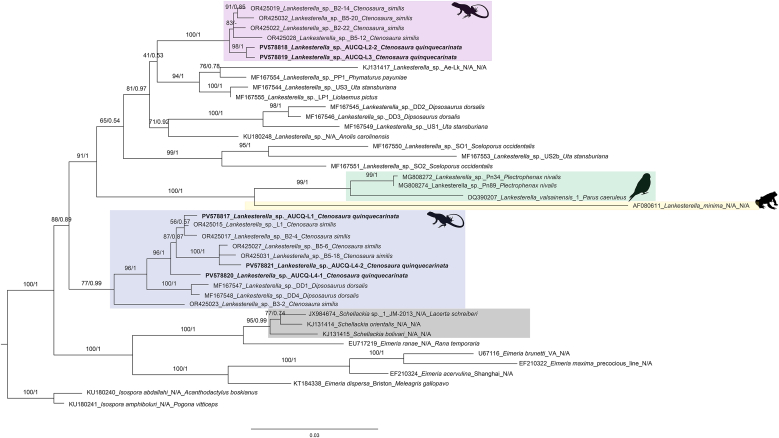
Phylogenetic analysis of *Lankesterella* Labbé (1894) based on 1481-bp 18S rDNA sequences. Sequences obtained in this study are highlighted in bold font. The 18S rDNA sequences from *Isospora* Schneider (1881) and *Eimeria Schneider*, 1875 were used as outgroups. Bootstrap values and posterior probabilities are shown close to the nodes. Sequence labels follow the format: accession number_scientific name_haplotype/clone_host species. N/A = Not available in the GenBank source modifier fields. The silhouettes located near the clades indicate the hosts from which the parasites were isolated: a frog for amphibians, a lizard for reptiles (pink and blue shades), and a bird for avian. Bayesian inference (BI) and Maximum Likelihood (ML) analyses recovered all newly generated haplotypes from *Ctenosaura quinquecarinata* (Gray, 1842) within the *Lankesterella* clade together with reference sequences. Three haplotypes (PV578817, PV578820, PV578821) clustered with *Lankesterella* haplotypes DD1 and DD4 from *Dipsosaurus dorsalis* (Baird and Girard, 1852) (MF167547, MF167548) and with haplotypes from *C. similis* (Gray, 1831) with strong support (ML bootstrap/posterior probability = 96/1). The remaining two haplotypes (PV578818, PV578819) formed another subclade with four C. similis haplotypes (ML bootstrap/posterior probability = 100/1). This Ctenosaura clade is sister to DD2 and DD3 from the same D. dorsalis individual as DD1/DD4 (MF167545, MF167546) and to additional Lankesterella sequences from diverse lizard hosts and localities. *Lankesterella* sequences from amphibians (yellow shade) and birds (green shade), and sequences of *Schellackia* Reichenow (1919) (gray shade) are also highlighted in the tree.

**Fig. 10:**
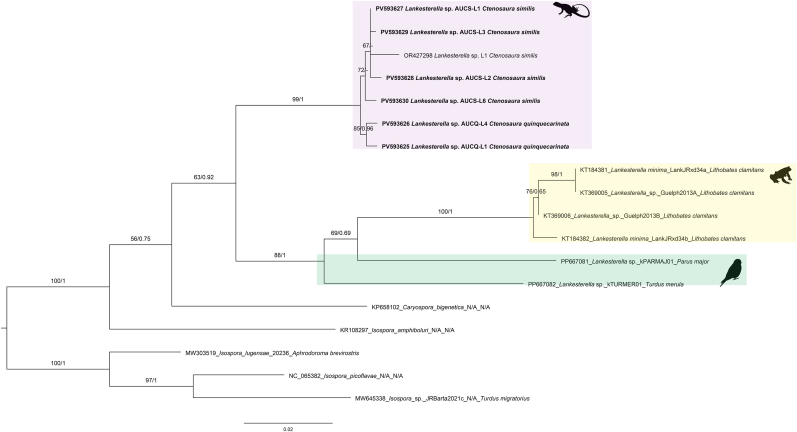
Phylogenetic analysis of *Lankesterella* Labbé (1894) based on 851-bp cytochrome oxidase c subunit I (*COI*) gene sequences. Sequences obtained in this study are highlighted in bold font. The *COI* sequences from *Isospora* Schneider (1881) and *Eimeria Schneider*, 1875 were used as outgroups. Bootstrap values and posterior probabilities are shown close to the nodes. Sequence labels follow the format: accession number_scientific name_haplotype/clone_host species. N/A = Not available in the GenBank source modifier fields. The silhouettes located near the clades indicate the hosts from which the parasites were isolated: a frog for amphibians, a lizard for reptiles, and a bird for avian. Bayesian inference (BI) and Maximum Likelihood (ML) analyses recovered all new sequences from *Ctenosaura quinquecarinata* (Gray, 1842) in a strongly supported monophyletic clade together with a previously reported *Lankesterell*a sequence from C. similis (Gray, 1831) (GenBank accession number: OR427298) (ML bootstrap/posterior probability = 99/1). *Lankesterella* sequences from amphibians (yellow shade) and birds (green shade) are also highlighted in the tree.

Partial 18S rDNA fragments of *Lankesterella* were successfully amplified from all five *C. quinquecarinata* individuals (GenBank accession numbers: PV578817–PV578821). Molecular cloning was performed for CQ2 because double peaks were observed in the chromatograms. Phylogenetic analysis recovered all newly generated haplotypes within the *Lankesterella* clade, together with reference sequences from earlier studies (Fig. 9). Three haplotypes (GenBank accession numbers: PV578817, PV578820, and PV578821) clustered with *Lankesterella* haplotypes DD1 and DD4 (GenBank accession numbers: MF167547 and MF167548) from the *Dipsosaurus dorsalis* (Baird and Girard, 1852) and with haplotypes from *C. similis* with strong nodal support (ML bootstrap/posterior probability = 96/1). Within this cluster, uncorrected pairwise distances ranged from 0.003 to 0.013, whereas distances to the remaining *C. quinquecarinata* haplotypes (e.g., PV578818) ranged from 0.032 to 0.038 (Supplementary Table S5). The remaining two haplotypes (GenBank accession numbers: PV578818 and PV578819) formed another subclade with four *C. similis* haplotypes (ML bootstrap/posterior probability = 100/1), and showed the closest p-distance (0.005) to haplotype from *C. similis* OR4250190. This Ctenosaura clade is sister to DD2 and DD3 from the same D. dorsalis individual as DD1/DD4 (MF167545, MF167546) and to additional *Lankesterella* sequences from diverse lizard hosts and localities (Fig. 9) (Supplementary Table S5). As reported in previous studies, all sequences of *Schellackia* sp., the hemococcidian species identified in Old World lizards, formed a separate monophyletic group, distinct from the *Lankesterella* clade. Partial *COI* sequences of hemococcidian parasites were amplified from C. quinquecarinata specimens CQ1 and CQ4 (GenBank accession numbers: PV593625 and PV593626), and from four of six *C. similis* specimens previously collected (Chang et al., 2023) (GenBank accession numbers: PV593627 to PV593630), yielding six new haplotypes. Pairwise uncorrected pairwise distances among these six haplotypes were 0.001–0.008, and their distances to the *Lankesterella* sequence from *C. similis* (OR427298) were 0.006–0.011 (Supplementary Table S6). Phylogenetic analysis placed all new sequences in a strongly supported monophyletic clade together with a previously reported *Lankesterella* sequence from *C. simili*s (GenBank accession number: OR427298) (ML bootstrap/posterior probability = 99/1) (Fig. 10). This clade was sister to a group comprising avian-associated *Lankesterella* (GenBank accession numbers: PP667081 and PP667082) and four haplotypes from *Lithobates clamitans* (Latreille, 1801) (GenBank accession numbers: KT184381, KT184382, KT369005, and KT369006).

#### *3.3.2* *C. similis* (CS1–CS2)

Partial 18S rDNA fragments of *Lankesterella* were amplified the *C. similis* (CS2). However, the sequence was excluded owing to poor chromatogram quality and failure of molecular cloning.

#### *3.3.3* *Basilisus plumifrons* (BP)

**Fig. 11:**
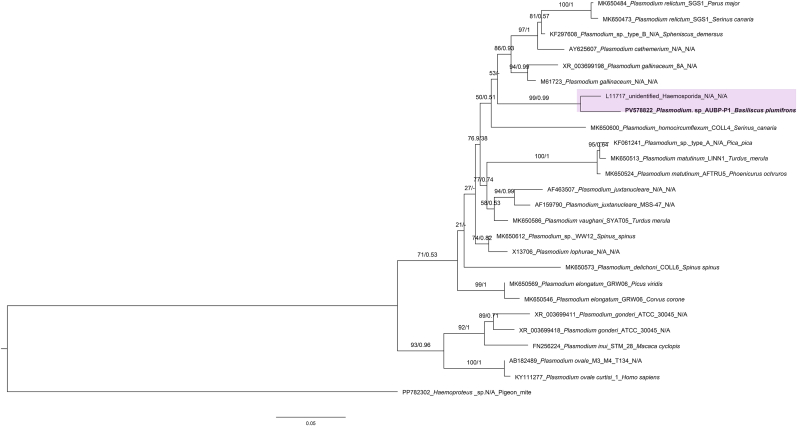
Phylogenetic analysis of *Plasmodium* Marchiafava and Celli (1885) based on 560-bp 18S rDNA sequences. Sequences obtained in this study are highlighted in bold font. The 18S rDNA sequence from *Haemoproteus* Kruse (1890) was used as outgroups. Bootstrap values and posterior probabilities are shown close to the nodes. Sequence labels follow the format: accession number_scientific name_haplotype/clone_host species. N/A = Not available in the GenBank source modifier fields. Bayesian inference (BI) and Maximum Likelihood (ML) analyses recovered the sequence obtained from the *Basiliscus plumifrons* Cope 1875 specimens (GenBank accession numbers: PV578822) in a strongly supported clade with an unclassified hemosporidian sequence (GenBank accession numbers: L11717) (ML bootstrap/posterior probability = 99/0.99) (pink shade).

**Fig. 12:**
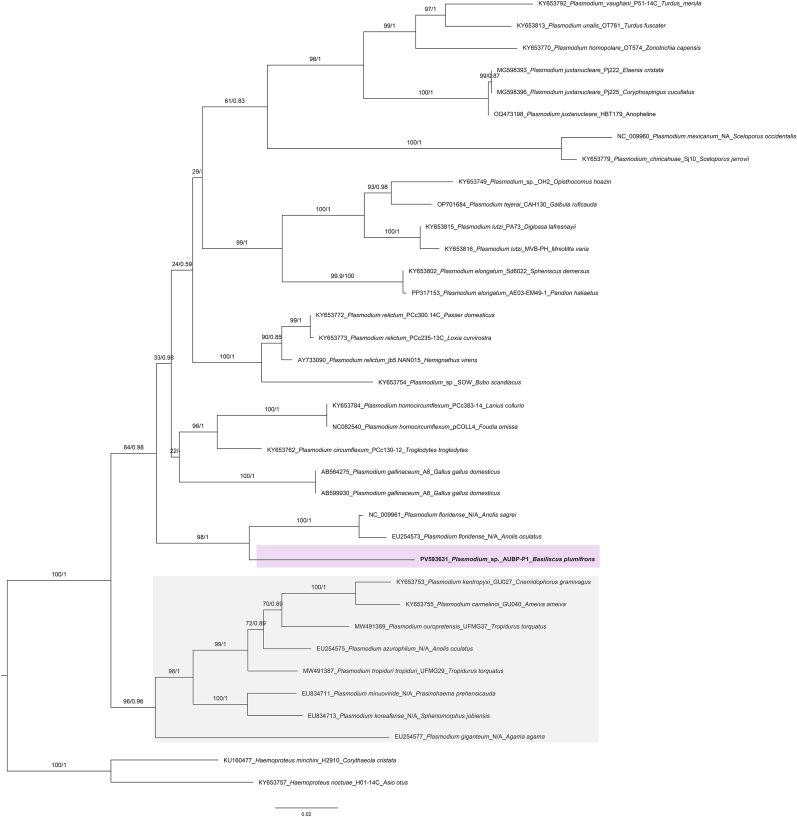
Phylogenetic analysis of *Plasmodium* Marchiafava and Celli (1885) based on 851-bp cytochrome oxidase c subunit I (*COI*) sequences. Sequences obtained in this study are highlighted in bold font. The *COI* sequences from *Haemoproteus* Kruse (1890) were used as outgroups. Bootstrap values and posterior probabilities are shown close to the nodes. Sequence labels follow the format: accession number_scientific name_haplotype/clone_host species. N/A = Not available in the GenBank source modifier fields. Bayesian inference (BI) and Maximum Likelihood (ML) analyses recovered the sequence obtained from *Basiliscus plumifrons* Cope 1875 in this study (GenBank accession numbers: PV593631) as a distinct, well-supported terminal branch (ML bootstrap/posterior probability = 98/1) within a clade comprising two *Plasmodium floridense* Thompson and Huff, 1944 sequences from *Anolis* Daudin (1802) (Linnaeus, 1758) (NC_009961 and EU254573) (pink shade). This clade did not nest with the South American lineages represented by *Plasmodium tropiduri* Aragão and Neiva, 1909, *Plasmodium ouropretensis* Córdoba et al. (2021) from *Tropidurus torquatus* (Wied-Neuwied, 1820), *Plasmodium carmelinoi*Lainson, 1960 from *Ameiva ameiva* (Linnaeus, 1758), and *Plasmodium kentropyxi* Lainson, 1960 from *Cnemidophorus gramivagus* McCrystal and Dixon, 1987 (gray shade).

Screening with the HepF300/Hep900 primer set also amplified an 18S rDNA fragment from the *B. plumifrons* specimens (GenBank accession numbers: PV578822). Phylogenetic analysis recovered this sequence in a strongly supported clade with an unclassified hemosporidian sequence (GenBank accession numbers: L11717) (ML bootstrap/posterior probability = 99/0.99) (Fig. 11). Consistently, uncorrected pairwise distances of PV578822 to *Plasmodium* sequences in the dataset ranged from 0.034 (to L11717) to 0.119 (Supplementary Table S7). Phylogenetic analysis of *COI* fragment recovered the sequence obtained from *B*. *plumifrons* in this study (GenBank accession numbers: PV593631) as a distinct, well-supported terminal branch (ML bootstrap/posterior probability = 98/1) within a clade comprising two *Plasmodium floridense* Thompson and Huff, 1944 sequences from *Anolis* Daudin (1802) (Linnaeus, 1758) (NC_009961 and EU254573) (Fig. 12). This clade did not nest with the South American lineages represented by *Plasmodium tropiduri* Aragão and Neiva, 1909 and *Plasmodium ouropretensis* Córdoba et al. (2021) from *Tropidurus torquatus* (Wied-Neuwied, 1820); *Plasmodium carmelinoi* Lainson, 1960 from *Ameiva ameiva* (Linnaeus, 1758); and *Plasmodium kentropyxi* Lainson, 1960 from *Cnemidophorus gramivagus* McCrystal and Dixon, 1987. The uncorrected pairwise distances of PV593631 to NC_009961 and EU254573 were 0.067 and 0.072, respectively, and 0.085 to 0.112 to the remaining *Plasmodium* sequences (Supplementary Table S8).

#### *3.3.4* *Stigmochelys pardalis* (SP)

Across phylogenetic trees inferred from both the 545-bp and 1515-bp 18S rDNA datasets, the sequence from *Stigmochelys pardalis* (PV578816) formed a clade with two *Hepatozoon fitzsimonsi Dias 1953* sequences from the same host species (PP718267 and PP718268) with strong support (ML bootstrap/posterior probability = 99/0.96 and 98/1 for the 545-bp and 1515-bp trees, respectively) (Fig. 2, Fig. 3). Consistently, the uncorrected pairwise distance among these three sequences across the aligned regions was 0.000 (Supplementary Tables S1 and S2).

## 4 Discussion

In this study, we investigated the hemoparasite infections in various genera of imported reptiles in Taiwan through an integrative approach combining clinical pathology, molecular biology, and histopathology.

The *V. macraei* individual examined in this study presented with marked emaciation, likely resulting from multiple concurrent infections, including a high intensity of *Hepatozoon infection*. Histopathological examination of the liver revealed prominent melanomacrophage center hyperplasia, consistent with an activated immune response (Jacobson et al., 2020). Although overt inflammatory cell infiltration was not observed around the parasites in the examined tissues, this may reflect the parasite life-cycle stage at the time of sampling—potentially prior to meront rupture and merozoite release (Wozniak et al., 1996). In the 565 bp 18S rDNA fragment dataset, our sequence PV578808 was highly divergent from all named *Hepatozoon* species and clustered with two unnamed varanid-associated *Hepatozoon* lineages. In 1515 bp 18S rDNA fragment datasets, our sequence formed a distinct, well-supported terminal branch within the reptilian *Hepatozoon* clade, consistent with its uncorrected pairwise distance profile. Although relationships between PV578808 and other varanid *Hepatozoon* could not be resolved owing to the paucity of sufficiently long sequences, the available molecular evidence indicates substantial divergence from described *Hepatozoon* species. Taken together with the morphological data, these findings suggest a novel or previously unrecognized species; we therefore propose the designation *Hepatozoon macraei* sp. nov. This underscores the necessity of acquiring longer and higher-resolution molecular markers, alongside detailed morphological observations, to clarify the diversity and evolutionary relationships of *Hepatozoon* species infecting reptiles (Sloboda et al., 2007; Vilcins et al., 2009; Úngari et al., 2021; Junsiri et al., 2024).

During this investigation, gamonts observed in *C. quinquecarinata* specimens showed a nearly uniform, elongated appearance with a relatively large nucleus paralleling the long axis of the parasites. These features are entirely distinct from the *H*. *gamezi*, which infects *C. similis* and showed sexual dimorphism, despite the close taxonomic relationship between *C. quinquecarinata* and *C. similis* (Desser, 1997; Chang et al., 2023). In the 18S rDNA phylogeny analysis, the sequences obtained from the *C. quinquecarinata* were genetically distant to the *H. gamezi* clade and clustered instead with the *H. ophisauri* sequence from the *P*. *apodus* in Iran. The uncorrected pairwise distances were also consistent with the phylogenetic result. Although many previous studies have suggested that *Hepatozoon* species possess broad host ranges, with the capacity to infect multiple host species (Sloboda et al., 2007; Maia et al., 2011), in the present study, we identified morphologically and phylogenetically distinct *Hepatozoon* infections in two closely related species, *Ctenosaura similis* and *C. quinquecarinata*. In the blood films of 4 *C. quinquecarinata*, the sporozoites with refractile bodies, a distinctive morphological characteristic compatible with those in the hemococcidian genera *Lankesterella* and *Schellackia,* were observed with different intensities (Paperna and Martin, 2001; Lemgruber and Lupetti, 2012). The morphological and morphometric data reveal high similarity with the *Lankesterella* sp. we identified in imported *C*. *similis* individuals (Chang et al., 2023). In both 18S rDNA and *COI* phylogenetic trees, these newly obtained amplicons are placed with the *Lankesterella* clade but also anchored in the clade formally composed of sequences of *Lankesterella* sp. from the *C. similis* specimens, with the uncorrected pairwise distance <0.01 in both 18S rDNA and *COI* datasets. Therefore, we suggested that the *C. quinquecarinata* were infected with the same hemococcidian in the *C. similis*, whose host range has now been redefined to at least 2 *Ctenosaura* species. In contrast to the host-specialist pattern of *Hepatozoon* infection in *Ctenosaura* species, *Lankesterella* sp. exhibits a host-generalist pattern; together, these findings highlight gaps in our understanding of parasite genetic diversity and host–parasite relationships.

Although necropsy and histopathological examination were not performed on the *B*. *plumifrons* in this study, clinical signs of emaciation, dehydration, and severe anemia documented on hematology were consistent with prior reports (Walden et al., 2020; Braga et al., 2025). In *Basiliscus basiliscus* (Linnaeus, 1758), infections with two *Plasmodium* species—*Plasmodium basilisci* (Pelaez and Pérez-Reyes, 1959) and *Plasmodium achiotense* (Telford, 1972)—have been reported (Telford, 1972). Morphologically, *P. achiotense* can be distinguished from *P. basilisci* by its prominently pigmented, large schizont containing 36 to 56 merozoites, as well as larger gametocytes (Telford, 1972). In our case, thin blood smears from the anemic and emaciated *B*. *plumifrons* contained trophozoites whose morphology more closely matched *P. achiotense*. However, we cannot confidently classify this parasite based solely on the limited morphological data available. Similar to the challenges faced in *Plasmodium* research among other reptiles, published records of *Plasmodium* infection in *Basiliscus* Laurenti, 1768 are based solely on historical morphometrics, with no sequence-based data added since the original description, hindering phylogenetic inference (Telford, 1972). Phylogenetic analysis of 18S rDNA fragment recovered that our sequence clustered with an unclassified hemosporidian lacking geographic and host metadata, and relationships to named reptile *Plasmodium* remained unresolved—likely reflecting limited phylogenetic signal in the short 18S fragment analyzed. Our *COI* phylogeny places the *Plasmodium* lineage recovered from the Central American *B*. *plumifrons* (PV593631) with the Caribbean/Central American *P. floridense* (NC_009961 and EU25457), rather than with South American lineages, a pattern supported by strong nodal values and pairwise distances. This phylogeographic pattern is concordant with prior work indicating the lizard *Plasmodium* species appear to be divided into three groups according to their geographic distribution (Perkins and Schall, 2002). Although our phylogenetic analyses did not yield conclusive results, to our knowledge we recorded the first *Plasmodium* sequence from *Basiliscus*, accompanied by morphometric data, providing a reference point for future studies of *Plasmodium* in *Basiliscus*. Additionally, a single parasite consistent with a *Plasmodium* meront was also observed within the cytoplasm of a thrombocyte. Previous studies indicate that *Plasmodium* infecting erythrocytes and thrombocytes within the same host or species belong to different species based on phylogenetic evidence (Matta et al., 2023). We did not recover additional divergent haplotypes, likely due to the extremely low parasitemia that impeded amplification. Nevertheless, the workflow established here provides a methodological platform for future surveys and studies of neglected reptile *Plasmodium* to further elucidate their diversity and potential impact on their hosts.

## 5 Conclusion

Although the exotic pet trade is flourishing worldwide, including in Taiwan, our understanding of the diseases affecting these species remains extremely limited. Reptiles host the greatest diversity of **hemoprotozoa**, yet many of these parasites remain poorly characterized or even undiscovered. Therefore, we recommend further research on the morphology, evolution, and effects of these parasites on their hosts, and emphasize the need for vigilant monitoring to detect any potential spillover into native reptile populations.

## CRediT authorship contribution statement

**Yen-Chi Chang:** Writing – original draft, Visualization, Methodology, Formal analysis, Data curation, Conceptualization. **Tai-Shen Lin:** Methodology, Investigation. **Wei-Wen Huang:** Resources, Methodology, Investigation. **Yi-Hsiang Huang:** Visualization, Resources, Methodology, Investigation, Data curation. **Cheng-Hsin Shih:** Validation, Supervision. **Ying-Chen Wu:** Writing – review & editing, Validation, Supervision, Resources. **Chiu-Chen Huang:** Writing – review & editing, Supervision, Resources. **Ter-Hsin Chen:** Writing – review & editing, Supervision, Resources.

## Declaration of competing interest

The authors declare that they have no known competing financial interests or personal relationships that could have appeared to influence the work reported in this paper.
