# Supplementary material for: Molecular and morphological characterization of hemoprotozoan infections in imported reptiles in Taiwan
Source: Int J Parasitol Parasites Wildl. 2025 Nov 17;28:101164. doi: 10.1016/j.ijppaw.2025.101164 (PMC12670453; doi:10.1016/j.ijppaw.2025.101164)
Supplement: Multimedia component 1 [file mmc1.docx]

Supplementary Fig. 1 Gamonts of *Hepatozoon macraei* sp. nov. Chang and Chen parasitising *Varanus macraei* – VP. Diff-Quik stain.

a) Low-power field of the blood smear. Three erythrocytic gamonts could be observed (arrows). Polychromasia is present in non-infected erythrocyte (arrowhead). bar = 50 µm. b-m) Immature gamonts were elongated, with unevenly dark-basophilic cytoplasm bearing 0-2 small eosinophilic granules and clumped and dense nuclei; the degree of dehemoglobulinization varied. b-d gamonts were curved with minimal tapering; f-h gamont were elongated and curved, tapered at both ends; i-k gamonts were gently sigmoid with ends recurved in opposite directions; l and m gamonts revealed wavy outline without cytoplasmic granules; nuclei are eccentrically placed toward the rounded end, and the opposite end was prominently tapered bar = 20 µm. n-p) Mature gamonts were robust; the cytoplasm was light basophilic to amphophilic without granules; and the nuclei were finely-stippled; the parasitophorous vacuole appeared as a sheath-like structures with a non-stained halo in the middle segment of each gamont (open arrows). bar = 20 µm
