# Supplementary material for: Molecular and morphological characterization of hemoprotozoan infections in imported reptiles in Taiwan
Source: Int J Parasitol Parasites Wildl. 2025 Nov 17;28:101164. doi: 10.1016/j.ijppaw.2025.101164 (PMC12670453; doi:10.1016/j.ijppaw.2025.101164)
Supplement: Multimedia component 2 [file mmc2.docx]

##### Primer pairs used in the ampliﬁcation reactions of apicomplexans (*Lankesterella* Labbé,1899 and *Hepatozoon* Miller 1908) DNA fragments based on the 18S rDNA gene and the mitochondrial cytochrome c oxidase subunit 1 gene (*COI*), references, and the size of ﬂanked fragments.

| Target gene | Primer | Sequence 5’-3’ | Temp | Fragment | Reference |
| --- | --- | --- | --- | --- | --- |
| 18S rDNA | BT-F1^a^  Hep1600R  EimIsoR1 | GGTTGATCCTGCCAGTAGT  AAAGGGCAGGGACGTAATCGG  AGGCATTCCTCGTTGAAGATT | 56 | 1600  1580 | (Megia-Palma et al., 2017) |
|  | HepF300^b^  Hep900^b^ | GTTTCTGACCTATCAGCTTTCGACG  CAAATCTAAGAATTTCACCTCTGAC | 56 | 600 | (Ujvari et al., 2004) |
| *COI* for *Lankesterella* | LankCOIF1  LankCOIR1 | CTGCTGCAAACCATAAAGAATTAGG  CAGGAATTCTACGTGGCATAACAT | 55 | 1200 | (Keckeisen et al., 2024) |
|  | LankCOIF2  LankCOIR2 | TGGTTCAGGTATTGGTTGGA  GACCATACTCTTAAGAATGGAGAATC | 52 | 900 | (Keckeisen et al., 2024) |

^a^ Common forward primer for Hep1600R and EimIsoR1

^b^ This primer set also amplified the *Plasmodium* Marchiafava & Celli 1885 18S rDNA sequence from the blood DNA of the *Basiliscus plumifrons* Cope 1875.

Abbreviation: Temp (^◦^C), annealing temperature.

##### Primer pair used in the ampliﬁcation reactions of hemosporidian DNA fragments based on the mitochondrial cytochrome c oxidase subunit 1 gene (*COI*), references, and the size of ﬂanked fragments.

| **Target gene** | **Primer** | **Sequence 5’-3’** | **Temp** | **Fragment** | **Reference** |
| --- | --- | --- | --- | --- | --- |
| ***COI*** | coIF  coIR | CTATTTATGGTTTTCATTTTTATTTGGTA  GTATTTTCTCGTAATGTTTTACCAAAGAA | 52 | 1300 | (Perkins and Austin, 2009) |

Abbreviation: Temp (^◦^C), annealing temperature.
