# Supplementary figures and images for: Molecular and morphological characterization of hemoprotozoan infections in imported reptiles in Taiwan
Source: Int J Parasitol Parasites Wildl. 2025 Nov 17;28:101164. doi: 10.1016/j.ijppaw.2025.101164 (PMC12670453; doi:10.1016/j.ijppaw.2025.101164)

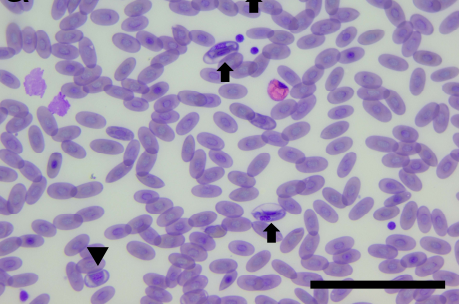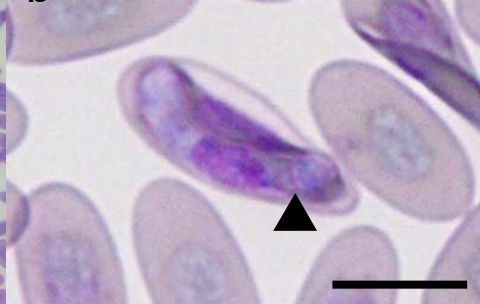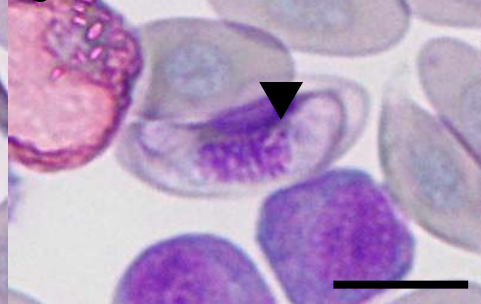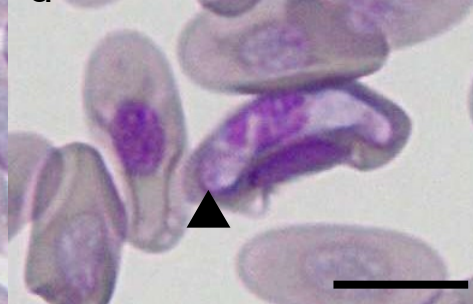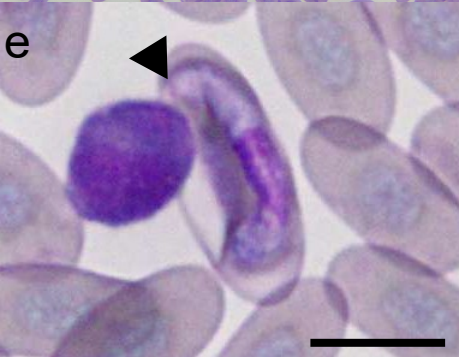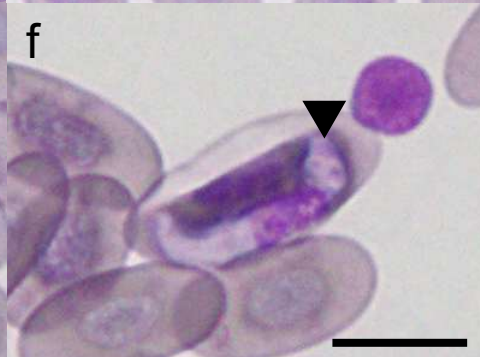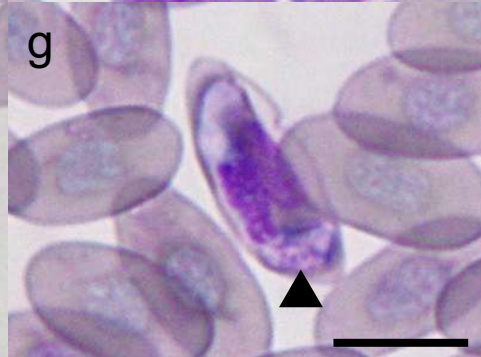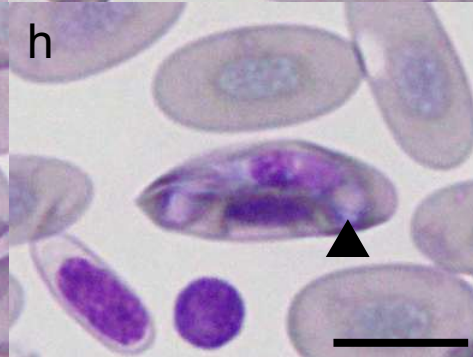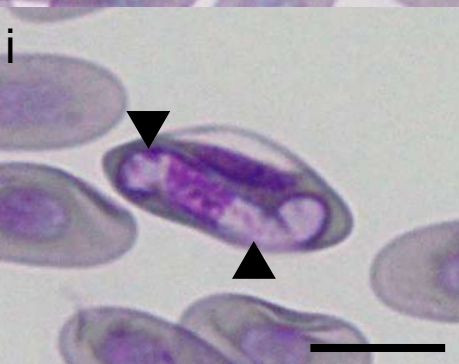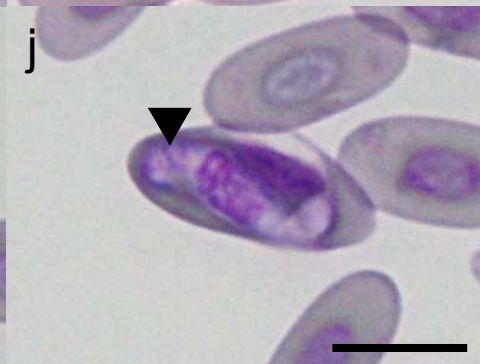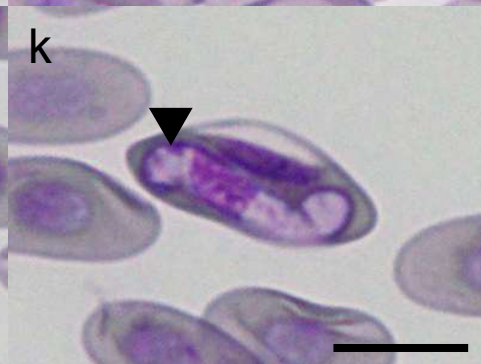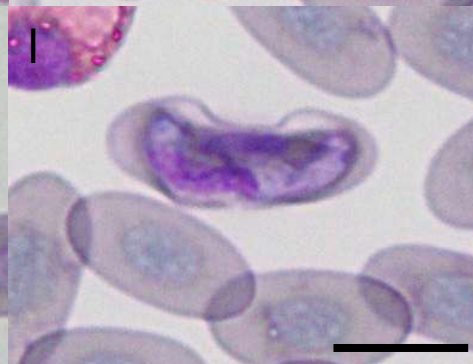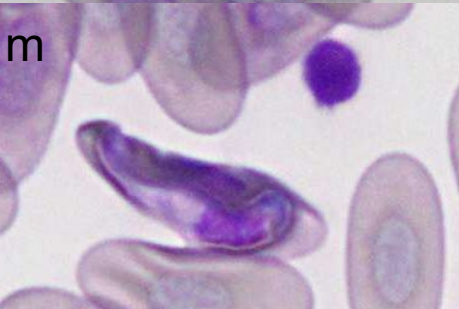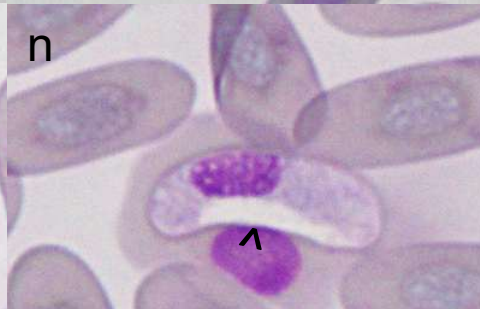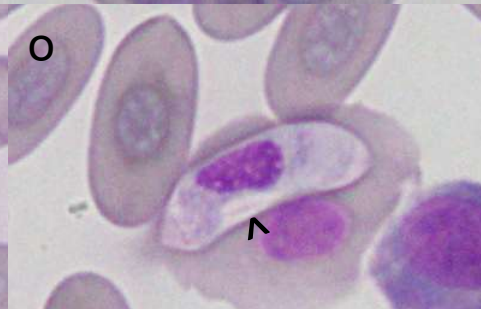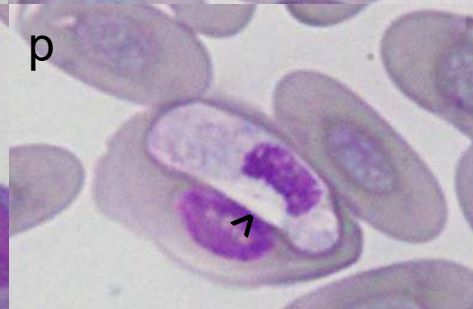

Supplement: Multimedia component 9 [file mmc9.pdf]
